# On the health effects of curcumin and its derivatives

**DOI:** 10.1002/fsn3.4469

**Published:** 2024-09-24

**Authors:** Hudda Ayub, Mahad Islam, Munnaza Saeed, Husnat Ahmad, Fahad Al‐Asmari, Mohamed Fawzy Ramadan, Mohammed Alissa, Muhammad Adnan Arif, Muhammad Umair Jamil Rana, Muhammad Subtain, Muhammad Abdul Rahim, Eliasse Zongo, Nazir Ahmad

**Affiliations:** ^1^ National Institute of Food Science & Technology University of Agriculture Faisalabad Pakistan; ^2^ Department of Food Science and Technology Muhammad Nawaz Shareef University of Agriculture Multan Pakistan; ^3^ Department of Food and Nutrition Sciences, College of Agricultural and Food Sciences King Faisal University Al‐Ahsa Saudi Arabia; ^4^ Department of Clinical Nutrition, Faculty of Applied Medical Sciences Umm Al‐Qura University Makkah Saudi Arabia; ^5^ Department of Medical Laboratory, College of Applied Medical Sciences Prince Sattam bin Abdulaziz University Al‐Kharj Saudi Arabia; ^6^ Department of Veterinary Sciences for Animal Health and Food Safety University of Turin Torino Italy; ^7^ Department of Food Science & Nutrition, Faculty of Medicine and Allied Health Sciences Times Institute Multan Pakistan; ^8^ Laboratoire de Recherche et d'Enseignement en Santé et Biotechnologies Animales Université Nazi BONI Bobo Dioulasso Burkina Faso; ^9^ Department of Nutritional Sciences Government College University Faisalabad Faisalabad Pakistan

**Keywords:** anti‐asthmatic, anti‐inflammatory, anti‐obesity, *Curcuma longa*, curcumin, diferuloylmethane, pharmacological activity

## Abstract

Turmeric (*Curcuma longa*) is an herbaceous plant that contains a phytochemical which is bright yellow and is known as curcumin. Turmeric, a member of Zingiberaceae family, has extensive application worldwide due to its beneficial medicinal attributes and is extensively used as a medicinal plant. Most people use turmeric as a spice, and it is a chief source of polyphenol curcumin. *Curcuma longa* has therapeutic properties, and since the initial extraction of curcumin from this plant, it has gained prodigious consideration from scientists in the medical field. The biological properties of curcumin, also known as 1,7‐bis (4‐hydroxy‐3‐methoxyphenyl)‐1,6‐heptadiene‐3,5‐dione, or diferuloylmethane, include anti‐inflammatory, anti‐oxidant, anti‐cancer, anti‐asthmatic, anti‐arthritic, neuroprotective, anti‐diabetic, anti‐obesity, wound‐healing, hepatoprotective, skin curative, reproductive role, etc. This work has reviewed many clinical trials and their findings about these activities. The focus of this review manuscript is concentrated on the presently existing clinical and animal studies, which exposed the possible anti‐retroviral activities of curcumin and its by‐products.

## Graphical abstract

*Curcuma longa* has therapeutic properties that have gained prodigious consideration from scientists in the medical field—review of the existing clinical and animal studies of curcumin and its by‐products. The biological properties of curcumin, also known as 1,7‐bis (4‐hydroxy‐3‐methoxyphenyl)‐1,6‐heptadiene‐3,5‐dione, or diferuloylmethane, include anti‐inflammatory, anti‐oxidant, anti‐cancer, anti‐asthmatic, anti‐arthritic, neuroprotective, anti‐diabetic, anti‐obesity, wound‐healing, hepatoprotective, skin curative, reproductive role, etc.

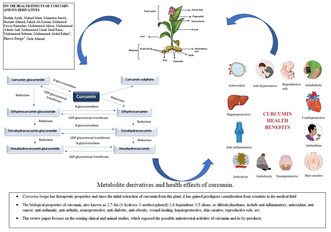

## 1 INTRODUCTION

**FIGURE 1:**
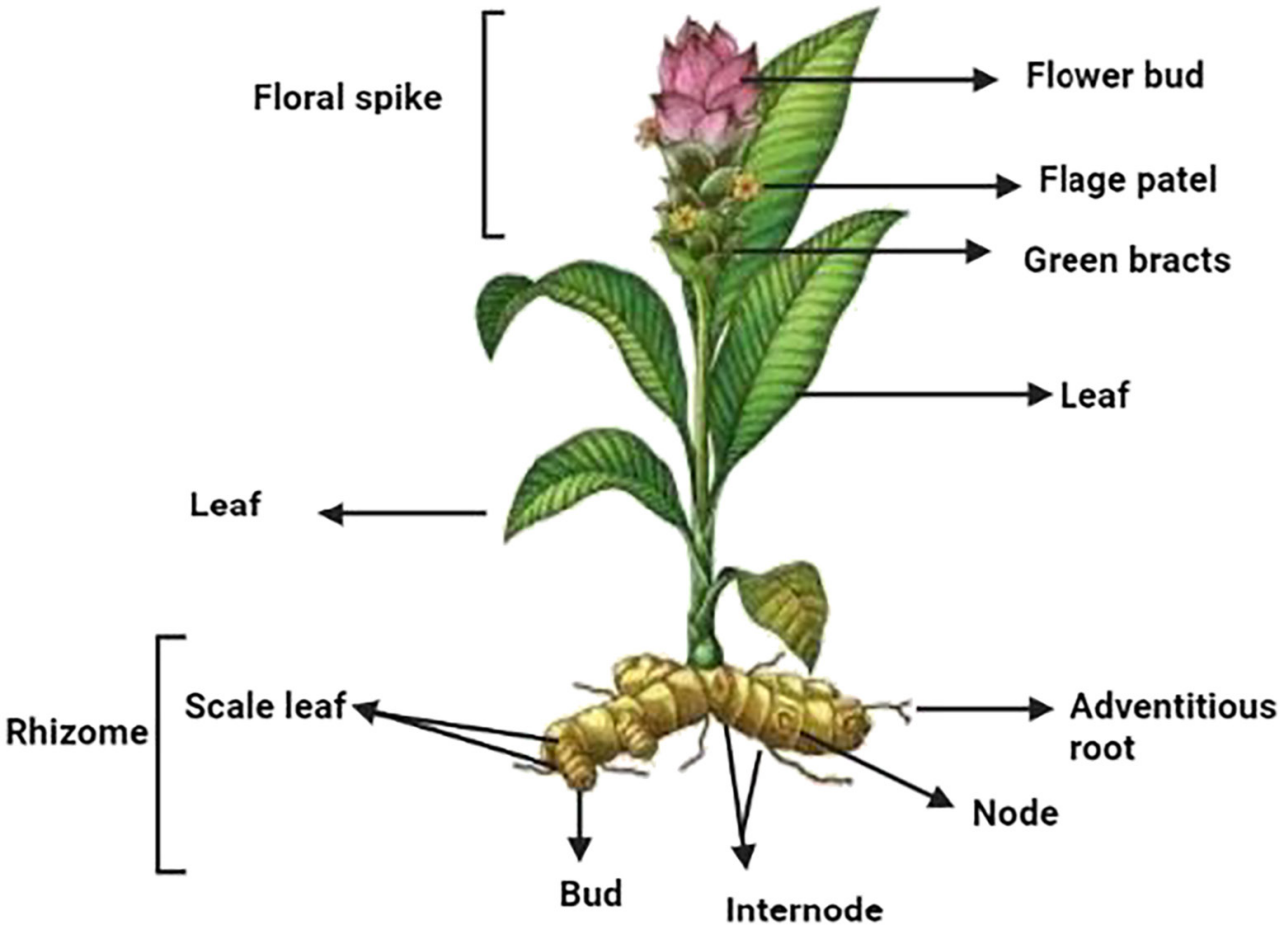
The diagram of the entire curcumin plant.

As long as humankind has existed, we have been using natural products for medicinal purposes for various diseases affecting health (Figure 1) (Carlson, 2010). A natural product indicates a chemical substance that comes from extraction or isolation from living organisms (Li, Gao, et al., 2016; Li, Larregieu, et al., 2016). The production of novel emerging medications from natural products is considered to be a perplexing task still requiring more efforts, and this operation occurs through the gathering, isolation, and purification, followed by characterization of these products, and the last step is the analysis of its toxicological‐and pharmacology‐related effects. But even after all these steps, bio‐products are considered a foundation of many chemical composites that have very novel structures and modes of action (Thomford et al., 2018).

A seasoning that has drawn significant interest from the domains of medicine and science along with the culinary world, turmeric (scientific name *Curcuma longa*), is an herbaceous plant that is rhizomatous and perennial in nature and a member of ginger family (Priyadarsini, 2014). For almost thousand years, turmeric's capabilities in the field of medicine have been known, but the capability of analyzing the exact science behind the action and bioactive compounds has just been studied recently (Gupta et al., 2013).

Curcumin, also known as diferuloylmethane, is a significant polyphenol found in the rhizome of *Curcuma longa* (commonly known as turmeric) and additional *Curcuma* species. It is 1,7‐bis(4‐hydroxy‐3‐methoxyphenyl)‐1,6‐heptadiene‐3,5‐dione. Traditionally, Asian countries have employed the herb *Curcuma longa* as a medicine (Mirhadi et al., 2024). Being the prime basis of curcumin, turmeric is thought to be the utmost considered herbal species. It also has a renowned antiquity of its use in traditional Chinese and Indian (Ayurveda) remedies for a variety of beneficial operations (Gupta et al., 2013). Curcumin is a phytochemical which is brightly yellow colored and is extracted from *Curcuma longa* rhizome belonging to Zingiberaceae. *Curcuma longa* also includes the curcuminoids bis‐desmethoxycurcumin and desmethoxycurcumin in addition to curcumin (Hewlings & Kalman, 2017). It is available in the markets in various forms likes tablets, ointments, and capsules (Chuengsamarn et al., 2012). Food and Drug Administration (FDA) has sanctioned the Curcuminoids as Generally Recognized as Safe (GRAS) (Akbik et al., 2014). Many results have directed that curcumin is harm free, and even at precise great prescriptions, it is tolerizeable (Lao, Demierre, et al., 2006; Lao, Ruffin, et al., 2006). In spite of its significant effects on pharmacology and safety, as a drug it has not been approved, and a potential reason has been justified by the bioavailability of curcumin, thus reducing translation into clinical conditions of in vitro advantages (Anand et al., 2007; Shakeri & Sahebkar, 2016). The extensive research on curcumin reveals notable differences between its effectiveness observed in vitro and in vivo. Despite its potential observed in vitro, curcumin encounters issues like poor water solubility, chemical instability, and low bioavailability, leading to reduced blood concentrations following oral administration (Nelson et al., 2017). These challenges restrict its therapeutic potential in humans. Amidst these obstacles, Nelson et al. (2017) further propose that curcumin could provide advantages via non‐systemic pathways, like influencing the gut flora, but data on this point are presently limited. Furthermore, due to possible synergistic effects and a wider pharmacological profile, holistic treatments utilizing crude turmeric extracts, which contain a mixture of curcuminoids and other compounds, may be more advantageous than pure curcumin.

## 2 CHEMISTRY

**FIGURE 2:**
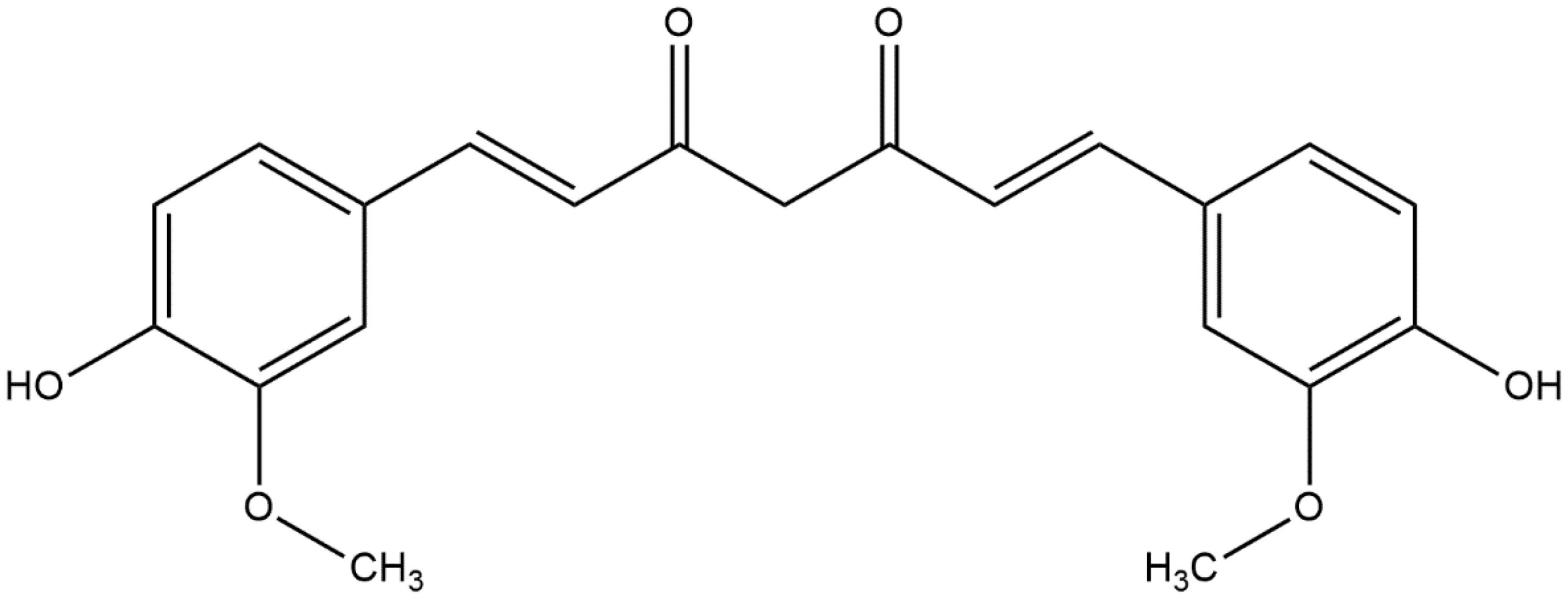
Chemical structure of curcumin.

Curcumin is termed as diferuloylmethane, a particle of regularity. This compound also has an International Union of Pure and Applied Chemistry (IUPAC) name (1E‐6E)‐1, 7‐bis (4‐hydroxy‐3‐methoxy phenyl)‐1, 6‐heptadiene‐3, 5‐dione. C_21_H_20_O_6_ is the biochemical formulae of curcumin, and 368.385 g/mole is its molar mass (Nelson et al., 2017). Three chemical substances are present in structure of curcumin: 2 oxy‐interchanged aryl moieties consisting of ortho‐methoxy phenolic (hydroxyl groups), which are joined through a chain of seven carbon having a α, β‐unsaturated β‐diketone moiety (Figure 2). Curcumin is regarded as the naturally going on analog that is most prevalent in a ratio of 60%–70% of a crude extract, which is then followed by demethoxycurcumin (DMC) in a ratio of 20%–30% and have one methoxy group missing, then comes the bisdemethoxycurcumin (BDMC) in a ratio of 10%–15% in which from both the aryl rings and the methoxy group are missing, and finally followed by many less occurring secondary metabolites (Priyadarsini, 2014). Curcumin serves as a potent anti‐oxidant and anti‐inflammatory agent (Lestari & Indrayanto, 2014), providing benefits such as pain relief, metabolic syndrome management (Kuptniratsaikul et al., 2014; Panahi et al., 2016), and improved kidney health (Trujillo et al., 2013). Additionally, curcumin exhibits anti‐mutagenic, anti‐microbial (Reddy et al., 2005), and anti‐cancer properties (Vera‐Ramirez et al., 2013). DMC shares similar therapeutic properties, with Hatamipour et al. (2019) suggesting it possesses anti‐inflammatory, neuroprotective, anti‐hypertensive, anti‐malarial, anti‐microbial, anti‐fungal, and vasodilatory properties. BDMC also displays anti‐inflammatory and anti‐oxidant properties (Ravindran et al., 2010) and has demonstrated potential in cancer treatment (Ramezani et al., 2018), although it is less extensively studied than curcumin and DMC. The physiological effects of curcumin, DMC, and BDMC involve modulating various molecular targets (Ahsan et al., 2020; Hatamipour et al., 2018; Ramezani et al., 2018). Metabolically, all three curcuminoids suffer from poor bioavailability (Liu et al., 2020; Zeng et al., 2007) though DMC and BDMC show slightly better bioavailability than curcumin (Desmarchelier et al., 2023), which is chemically instable and undergoes rapid metabolism (Metzler et al., 2013).

Curcumin's biological activity is related with the hydrogen‐atom reactions of donating, which leads to the curcumin oxidation. Important chemical reactions concerned with the curcumin's biological properties, also irreversible and reversible reactions of nucleophiles, are enzymatic reactions, degeneration, and hydrolysis (Nelson et al., 2017). All of them have a very substantial part in various organic attributes of curcumin. It is a water‐repelling molecule with a computed log *P* value of 3.43; however, it is insoluble in water, which indicates a weak network and biological availability (Kumar et al., 2016). However, it is dispersible in polar solvents such as ethanol, methanol (MeOH), acetone, and dimethylsulfoxide (DMSO) (Goel et al., 2008).

## 3 PHARMACOKINETIC PROPERTIES

Prior studies have depicted hurdles in attaining optimum concentrations for conditions of therapy of the molecule because of the reduced dispersibility and weakened biological availability of curcumin. Research results indicated that curcumin is firstly bio‐transformed into tetrahydrocurcumin and dihydrocurcumin and at last to conjugates of monoglucuronide. Preliminarily studies on animals described the fast metabolization of curcumin and its conjugation in liver, and, subsequently, with reduced systemic bioavailability, it is excreted in the feces. In different kinds of studies related curcumin's metabolism on rodents, excretion and biodistribution have been reported (Mirzaei et al., 2017).

The bioavailability and pharmacokinetic‐related studies of curcumin have shown results indicating its very low absorption in the intestines and speedy removal within the body (Anand et al., 2007). The overall results demonstrated that curcumin following oral use showed decreased absorption and fast extraction. In a preliminary project conducted, there was an administration to rats of curcumin with a dose of 1 g/kg that showed results in about 75% feces excretion, while very less amount was observed in urine (Sharma et al., 2007). Also, in another research, it was reported that curcumin absorption was 60% in rats after use via oral pathway (Gutierres et al., 2015). Results from a radio tracing investigation showed that curcumin undergoes transformation after intestinal absorption of 3 H‐radiolabeled curcumin (Sharma et al., 2007).

**FIGURE 3:**
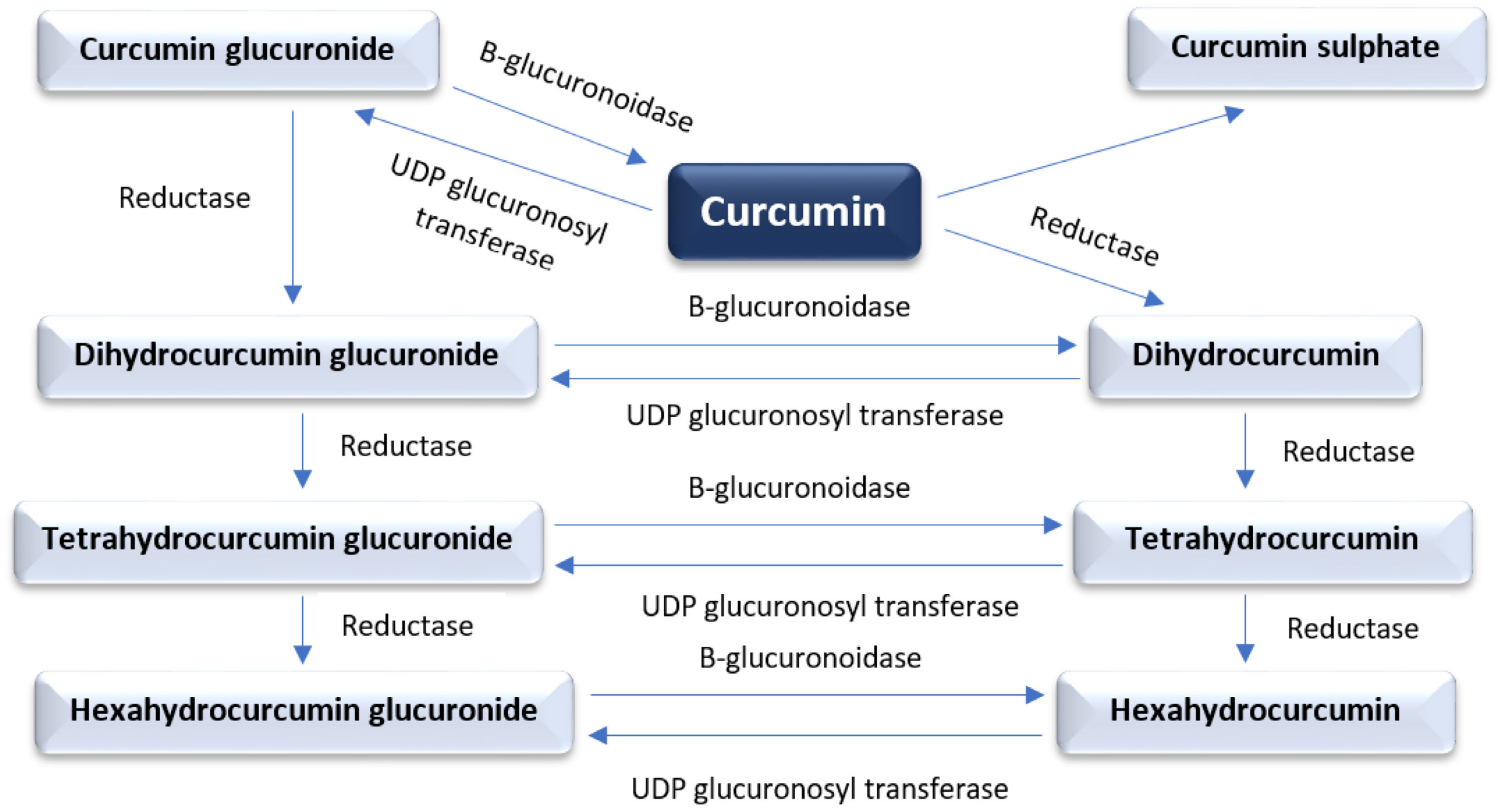
Metabolite derivatives of curcumin.

Many studies have reported that initially curcumin is converted to tetrahydrocurcumin and dihydrocurcumin via the action of reductases, followed by their conversion into monoglucuronide affix as tetrahydrocurcumin‐glucuronide and dihydrocurcumin‐glucuronide by the activity of β‐glucuronidase. The transformation actions of curcumin are shown in Figure 3. The change of the liver and intestines may be responsible for curcumin sulfates and curcumin glucuronides generation, or in contrast, it can also reduce molecules like hexahydrocurcumin (Pulido‐Moran et al., 2016).

Besides glucuronidation, a well‐established metabolic pathway for curcumin, it is also essential to consider other metabolic processes like sulfation. Sulfation, mediated by sulfotransferase (SULT) enzymes such as SULT1A3, 1B1, 1C4, and 1E1, results in the production of phase II metabolites, including glucuronides and sulfates. These metabolites account for the majority of circulating curcuminoids in both humans and animals (Lu et al., 2017). Curcumin undergoes phase II conjugation to produce both curcumin sulfate and curcumin glucuronide, while phase I bioreduction generates various reduced forms of curcumin. Notably, curcumin sulfate is a major metabolite among these products (Prasad et al., 2014; Stanić, 2017).

Gutierres et al. (2015) reported levels of curcumin in rat plasma. Also, they analyzed the insulin compassion variations and glucose forbearance in rats, which were streptozotocin‐diabetic when they were cured with yoghurt enriched with curcumin. The half‐life of curcumin's elimination was observed to be of 32.7 ± 12.9 (oral) and 8.6 ± 2.3 (IV) minutes. The bioavailability through the oral pathway was reported about 0.47%.

The time it took to attain the *C*_max_ (*t*_max_) in plasma of curcumin was reported to be 5 min (*C*_max_: 3.140.9 mg/mL; 10 mg/kg IV), and it was reported to be 14 min (*C*_max_: 0.060.01 mg/mL; 500 mg/kg oral) (Gutierres et al., 2015). Also, various studies have depicted a positive effect of complexation of phospholipid in boosting up the therapy effects of molecules with reduced absorption in oral pathway (Küllenberg et al., 2012). Silybin, if considered as an example, is a flavonolignan which is said to have low oral absorption values (Grattagliano et al., 2013). Studies revealed that silibinin which was phospholipidated showed an increased oral bioavailability and improved effects as anti‐oxidants and hepatoprotective effects in comparison with purified compound (Yanyu et al., 2006). One more study conducted with quercetin reported that phospholipidated composite of the quantified compound had a more intense therapeutic effect in contrast with the pure compound for prevention against injury of rat liver initiated by carbon tetra chloride (CCl_4_) (Maiti et al., 2005). According to the findings of another study, the absorption of a standardized mixture of curcuminoid and its phosphatidylcholine complex (lecithinized) formulation were comparable (Cuomo et al., 2011).

A basic procedure that has been used for boosting up the curcumin's bioavailability is the application of compounds that prevent curcumin's metabolic route activity. Study results of a project on methods of increasing curcumin's bioavailability reported that administration of piperine with oral curcumin, which is actually an alkaloid present in long pepper (*Piper longa*) and black pepper (*Piper nigrum*), found out to have an effect on improving the curcumin's serum concentrations in rodents. Additionally, piperine is a powerful inhibitor of glucuronidation of hepatic and intestines. Using increased doses of oral curcumin (2000 mg/kg) along with the piperine, the systemic bioavailability was found to be increased by 154% (Rathore et al., 2020). Various trials of phase I focused on cancer patients depicted information about pharmacokinetics, human bioavailability of curcumin, and metabolites.

Research conducted on almost 15 patients of colorectal cancer on advanced stage indicated even decreased concentrations of curcumin in serum. Doses of about 3.6 and 0.45 g were given on daily basis for 4 months in this research. From the six patients that were given 3.6 g dose in almost three patients, the average concentrations of plasma checked at all stages during the first month of the therapy trials were found out to be consistently 11.1 ± 0.6 nmol/L. In the patients administered with lower doses, curcumin was not found (Sharma et al., 2004). Because of the decreased curcumin's bioavailability, Theracurmin, which is an artificially extracted nanoparticle type of curcumin, was found having a sophisticated biological availability. Research looking at the pharmacological attributes of Theracurmin in patients without any disease attained acceptable concentrations of plasma after just one dose. Further studies exploring the well‐being of curcumin conducted on those with cancer depicted the same results. The other research, using a mouthful of curcumin every day with standard chemotherapy, was conducted based on gemcitabine. Maximum plasma curcumin concentrations (Median) following 200 mg Theracurmin prescription were found out to be 324 ng/mL, and for dose of 400 mg, Theracurmin maximum plasma level attained was 440 ng/mL, with no expected harmful activities observed during 9 months of research (Kanai et al., 2012). Table 1 shows curcumin concentration in biotic models through different routes of administration.

**TABLE 1:** Curcumin pharmacokinetic studies.

Route of administration	Dose	Species	Level	Tissue/plasma	Reference
Mouth	100.1 mg/kg	Mice	0.221 μg/mL	Plasma	Mirzaei et al. (2017)
I.P.	100.02 mg/kg	Mice	2.91 nmol/g 16.1 nmol/g 73.2 nmol/g 25.0 nmol/g 200.01 nmol/g	Brain Lungs Liver Plasma Intestinal mucosa	Mirzaei et al. (2017)
Oral	340 mg/kg	Rat	206 ng/g 807 ng/g 3671.8 ng/g 16.1 ng/mL 6.5 ng/g	Kidney Heart Liver Plasma Serum	Marczylo et al. (2009) Marczylo et al. (2007)
Mouth	1.1 g/kg	Rat	0.51 μg/mL	Serum	Maiti et al. (2007)
Mouth	500.03 mg/kg	Rat	0.059 μg/mL	Plasma	Yang et al. (2007)
Mouth	0.4–3.6 g	Human	12.7 ± 5.7 nmol/g	Colorectum	Garcea et al. (2005)
Oral	10–12 g	Human	50.51 ng/mL	Serum	Lao, Demierre, et al. (2006), Lao, Ruffin, et al. (2006)

### 3.1 Analytical methods

Analytical techniques are essential for quantifying and identifying both target compounds and impurities. A wide range of analytical methods for the qualitative and quantitative analyses of curcumin (CCM) across different matrices are well‐documented in the literature. These methods are vital for ensuring the quality control of both raw materials and finished products (Jurenka, 2009; Rohman, 2012).

Research interest in curcumin has surged as evidenced by the increasing number of citations. This interest is driven by curcumin's applications in pharmaceutical and drug delivery systems. Over the past decade, there has been a noticeable increase in the development of methodology for CCM analysis; nevertheless, there was a fall from 2014, which may have been brought on by the drive for green chemistry and more efficient, low‐resource techniques.

It is crucial to understand that different approaches can be used within a single technique, and the most appropriate path of action will rely on the needs and resources of the analyst. We go into further depth about some of the techniques offered to show the variety of approaches for CCM quantification.

### 3.2 UV–vis and fluorescence methods

UV–vis and fluorescence methods are quite advantageous when it comes to substance quantification because they are quick and simple to use. Compared to separation procedures, which need vast volumes of solvents and need more labor‐intensive processes, these methods are faster because they entail dissolving the sample in a solvent and measuring absorbance at particular wavelengths. However, when other materials in the matrix absorb at the same wavelength, their effectiveness may be reduced, which could lead to errors and problems with repeatability. Therefore, an initial examination of the matrix is necessary especially when using carriers that absorb in the same area (Jayaprakasha et al., 2005; Siddiqui et al., 2017).

When working with carriers, quantification is often expressed as entrapment efficiency (%EE) and loading capacity (%LC), which can be calculated using the equations below:

$$\%\mathrm{EE}=\left(\frac{\text{drug added‐free “unentrapped” drug}}{\text{drug added}}\right)\times 100$$

$$\%\mathrm{LC}=\left(\frac{\text{entrapped drug}}{\text{nanoparticles weight}}\right)\times 100$$

In order to reduce repeatability errors and facilitate the analysis of multiple samples, fluorescence and UV–vis techniques were additionally utilized in conjunction with flow injection analysis (Inoue et al., 2001; Thongchai et al., 2009).

### 3.3 Thin‐layer chromatography and high‐performance thin‐layer chromatography

For many years, both qualitative and quantitative analyses of a wide range of compounds have been conducted using thin‐layer chromatography (TLC) and high‐performance thin‐layer chromatography (HPTLC). When assessing unknown compounds in samples, these methods are more economical than high‐performance liquid chromatography (HPLC). HPTLC is favored over TLC because it can assess the chromatogram more thoroughly and permit a wider range of parameters without lengthening the analysis period. However, the accuracy of the detector can be compromised depending on the compound. Furthermore, since reverse‐phase reagents are scarce and costly, these methods are mainly employed in the normal phase (Loescher et al., 2014; Pozharitskaya et al., 2007; Siddiqui et al., 2017). The application of both methods for curcumin (CCM) quantification has been reported in numerous studies (Siddiqui et al., 2017; Zorofchian Moghadamtousi et al., 2014).

### 3.4 High‐performance liquid chromatography

HPLC is the most frequently used method for quantification due to its high precision, accuracy, and excellent limit of detection. However, these benefits come with significant costs related to solvents, instrumentation, and columns. In a study by Wichitnithad et al. (2009), a simple isocratic HPLC‐UV method was developed to simultaneously analyze three curcuminoids (curcumin, desmethoxycurcumin, and bisdesmethoxycurcumin) in turmeric extracts. The method, using reverse‐phase chromatography on a C18 column with isocratic elution, provided effective separation and quantification and was validated for accuracy and precision in routine quality control of turmeric extracts. C18 columns are commonly used because of the labile nature of curcuminoids (Jadhav et al., 2007). Additionally, the use of C8 columns has been reported (Kim et al., 2017).

Mobile phases for chromatography typically include MeOH and acetonitrile in proportions greater than 40%. Although acids are commonly used in chromatographic methods, research on their specific effects is limited. Trifluoroacetic acid is noted for enhancing resolution and improving peak shape (Cheng et al., 2010; Jadhav et al., 2007). Since each column has its unique pH range of analysis, it is crucial to note that pH must be maintained prior to analysis so as to avoid any degradation or change of the stationary phase and, subsequently, the compromise of the column (Wichitnithad et al., 2009).

Detectors commonly used with chromatographic systems are mass spectrometry (MS) and UV–vis. According to Cao et al. (2014) in both the positive and negative modes, LC–MS systems exhibit comparable detection limits (~1 ng/mL). UV–vis studies often use wavelengths around 425 nm, though some use 230 nm for simultaneous quantifications or due to instrumental limitations (Memvanga et al., 2014; Zhang et al., 2009).

### 3.5 Infrared spectroscopy

Infrared spectroscopy combined with chemometric tools is a promising method for quantifying substances due to its speed, minimal resource requirements, and ability to analyze multiple substances simultaneously (Lestari et al., 2017; Roggo et al., 2007; Rohman et al., 2010). Lestari et al. (2017) and Tanaka et al. (2008) used Fourier‐transform infrared and near‐infrared (NIR) spectroscopy to estimate curcuminoid content in plants, achieving results consistent with HPLC‐UV analysis. However, variations in sample composition or matrix effects can lead to inaccurate predictions, highlighting the need for system‐specific information to improve model accuracy.

## 4 HEALTH EFFECTS

**FIGURE 4:**
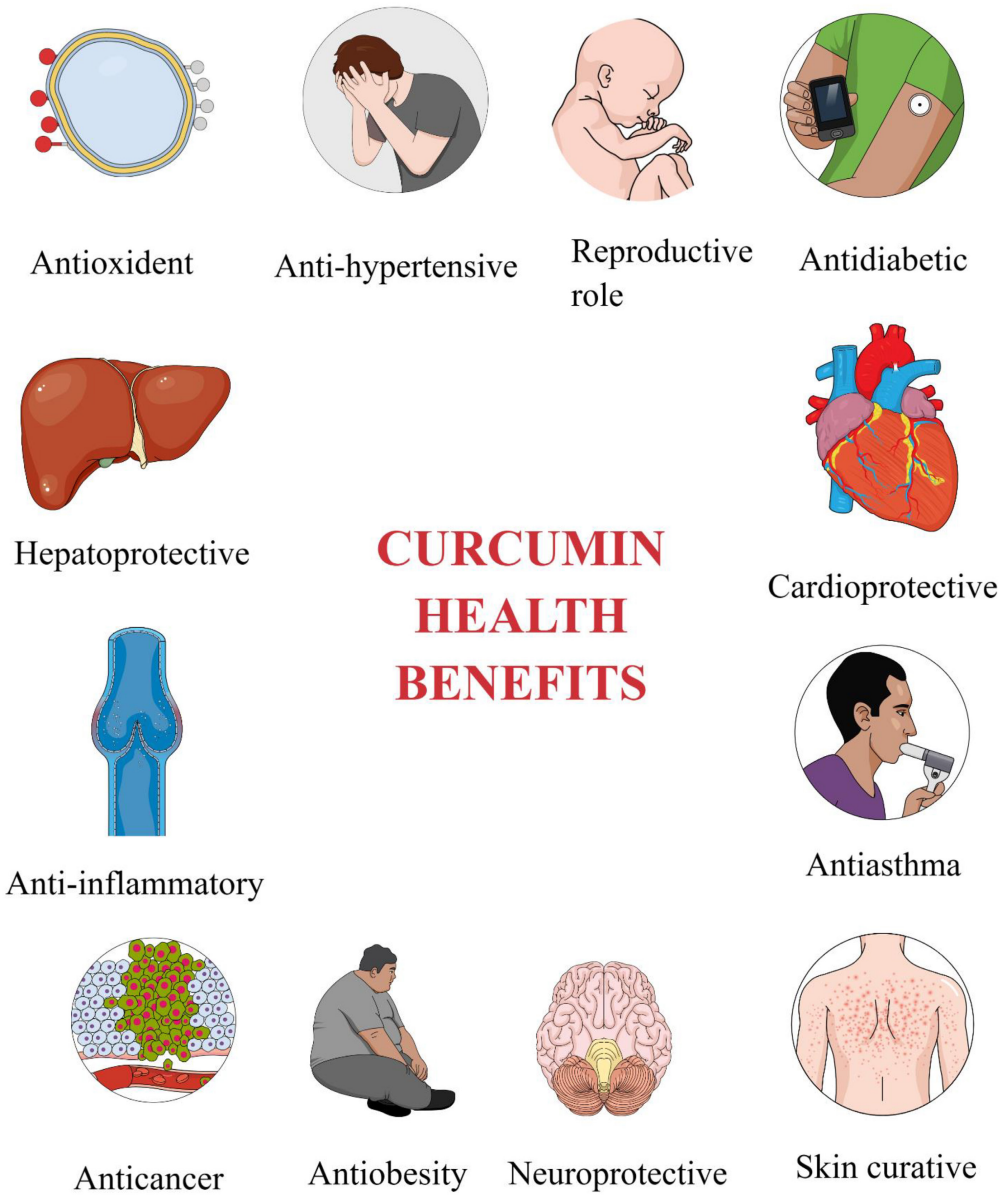
Health effects of curcumin.

Detailed information regarding health effects of curcumin is given below which were proved in the in vivo studies (Figure 4).

### 4.1 Anti‐oxidant activity

Oxidative stress results from an imbalance in the formation of reactive species, which are naturally generated in the organism's body and anti‐oxidants of endogenous nature. ROS, abbreviated as reactive‐oxygen species and formed naturally in the processes (cellular respiration), includes radicals like hydroxyl radical (OH), superoxide radical (O^2−^), hydrogen peroxide (H_2_O_2_), and singlet oxygen (O) (Gülçin, 2012). Disproportionate production of ROS can damage tissues. A human body prevents harm triggered by ROS via the anti‐oxidant defense system of its own and which consists of catalase (CAT) which is the reduced form of glutathione (GSH) and superoxide dismutase (SOD) (Batinić‐Haberle et al., 2010).

For oxidative stress, curcumin is identified as an improver of systemic markers, and it was found to be modulating the actions of GSH, SOD enzymes, and CAT that were responsible for the free radical neutralizations (Marchiani et al., 2014; Sahebkar et al., 2015). Using ferric thiocyanate, total anti‐oxidant activity was assessed after several in vitro studies of anti‐oxidants, including the 2,2′‐azino‐bis (3‐ethylbenzthiazoline‐6‐sulfonic acid) (ABTS) radical scavenging activity, the 1,1‐diphenyl‐2‐picrylhydrazyl free radical (DPPH) scavenging, and the superoxide anion radical scavenging using the ribofla (David et al., 2015).

This proved that curcumin could improve the serum actions of anti‐oxidants like SOD. A review and meta‐analysis of scattered control data showed that supplementation with pure curcuminoids had a significant effect on all studied parameters of oxidative stress, including plasma activities of CAT and SOD, glutathione peroxidase (GPx) in serum, and lipid peroxides (Banach et al., 2014). The impact of curcumin on free radicals is conducted using various kinds of mechanisms. It can remove several kinds of radicals, including reactive nitrogen and oxygen species (RNS and ROS, respectively) (Menon & Sudheer, 2007); it has the capability of modulating the actions of GSH, SOD enzymes, and CAT active in the process of free radical neutralization; also, it can suppress the enzymes responsible for generating ROS, like xanthine hydrogenase/oxidase and lipoxygenase/cyclooxygenase (Lin et al., 2007). Additionally, curcumin is a compound of lipophilic nature which differentiates it as a peroxyl radical scavenger, and that is why, just like vitamin E, curcumin is also said to have the properties of chain‐breaking anti‐oxidants and also considered as a chain‐breaking anti‐oxidant (Priyadarsini et al., 2003).

Ali et al. (2018) studied the influence of curcumin (diferuloyl methane, a turmeric pigment of phenolic nature), which has found to have strong anti‐oxidant, anti‐apoptotic, and anti‐inflammatory potential on the structure of kidney and its function in rats using adenine‐induced chronic kidney disease (CKD). The rats were administered for 5 weeks with adenine to initiate CKD‐like damage of kidney followed by three doses of curcumin. Markers that are used for kidney functioning indication and oxidative stress were enumerated for urine, renal homogenates, kidney tissue, and plasma. Curcumin shows productive impacts against adenine‐induced CKD by decreasing the inflammation and oxidative stress through the boosting up of transcription factor Nrf2.

**FIGURE 5:**
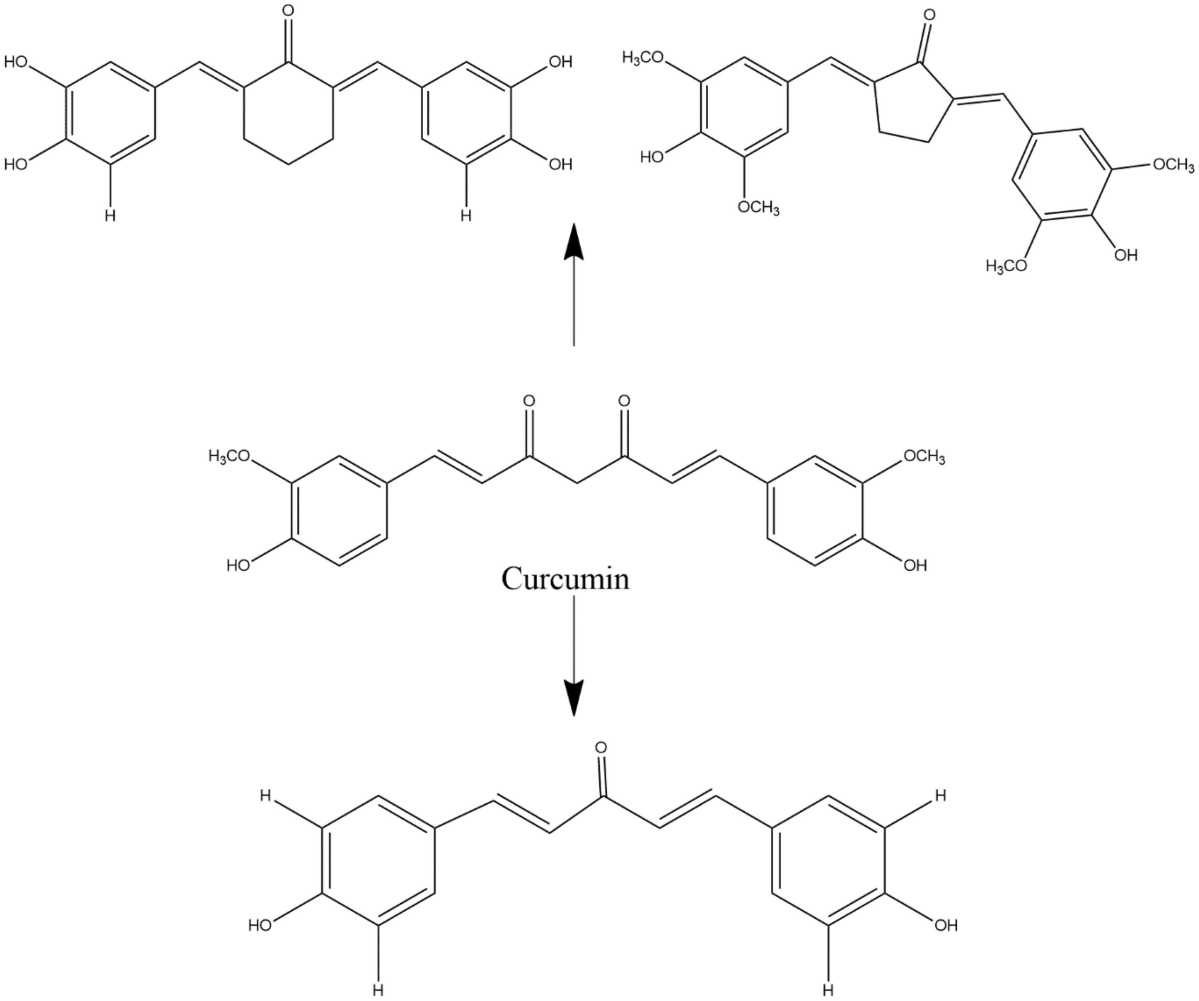
Curcumin derivatives have anti‐oxidant activity.

Numerous curcumin derivatives have been created during the past 20 years with the aim of discovering novel compounds with potent anti‐oxidant action. Three series of compounds of curcumin were created by Shang and his coworkers (Figure 5), and they analyzed their activities relating to anti‐oxidation in contrast to curcumin. Their results showed that the compounds consisting of O‐dimethoxy‐phenoxyl and O‐diphenoxyl groups implicated an increased anti‐oxidant activity compared to compounds without those groups. Additionally, they stated that the reduction of the compound to 5 carbon spacers significantly diminished the scavenging activity and that the 7‐carbon spacer is crucial for the scavenging activities. They also showed how lipophilicity, which is created by increasing the number of carbon atoms, is essential for anti‐oxidant activity (Shang et al., 2010).

### 4.2 Anti‐cancer activity

Almost one‐fifth of the deaths all over the world yearly are due to different kinds of cancers (Eric, 2012). It is considered the second‐largest reason of death taking over six million lives every year globally (Fu et al., 2015). Cancer occurs because of sequential genetic and epigenetic variations, which result in metastasis, angiogenesis, uncontrolled cell proliferation, and apoptosis (Hanahan & Weinberg, 2011).

Recently, curcumin's anti‐cancer activities have been investigated widely, and in reports, many considerable improvements have been observed in gastrointestinal, lung and breast cancers, genitourinary, and melanoma (Anand et al., 2008; Bar‐Sela et al., 2010; Ravindran et al., 2009). Several reports indicated anti‐cancer properties of curcumin as itself or mixed with other chemotherapy drugs in treating different kinds of cancer or other problems associated to cancer (Attari et al., 2015; Fiala, 2015; Wilken et al., 2011). Many studies on in vivo and in vitro have revealed that carcinogenesis can be prevented using curcumin by influencing two primary processes: tumor growth and angiogenesis (Rubagotti et al., 2017). Curcumin has shown affective anti‐fungal along with anti‐cancer properties when taken separately or with other drugs related to chemotherapy and anti‐fungal agents (Asti et al., 2014). Analogs of curcumin S1–S3, which have sulfone, effectively retard the growth of the human prostate, along with pancreatic, lung, and colon cancerous cells (Allegra et al., 2017; Stanić, 2017).

Curcumin has attributes to suppress the propagation and initiate apoptosis (programed cell death) in different cancer types, like:

- Breast cancer

**FIGURE 6:**
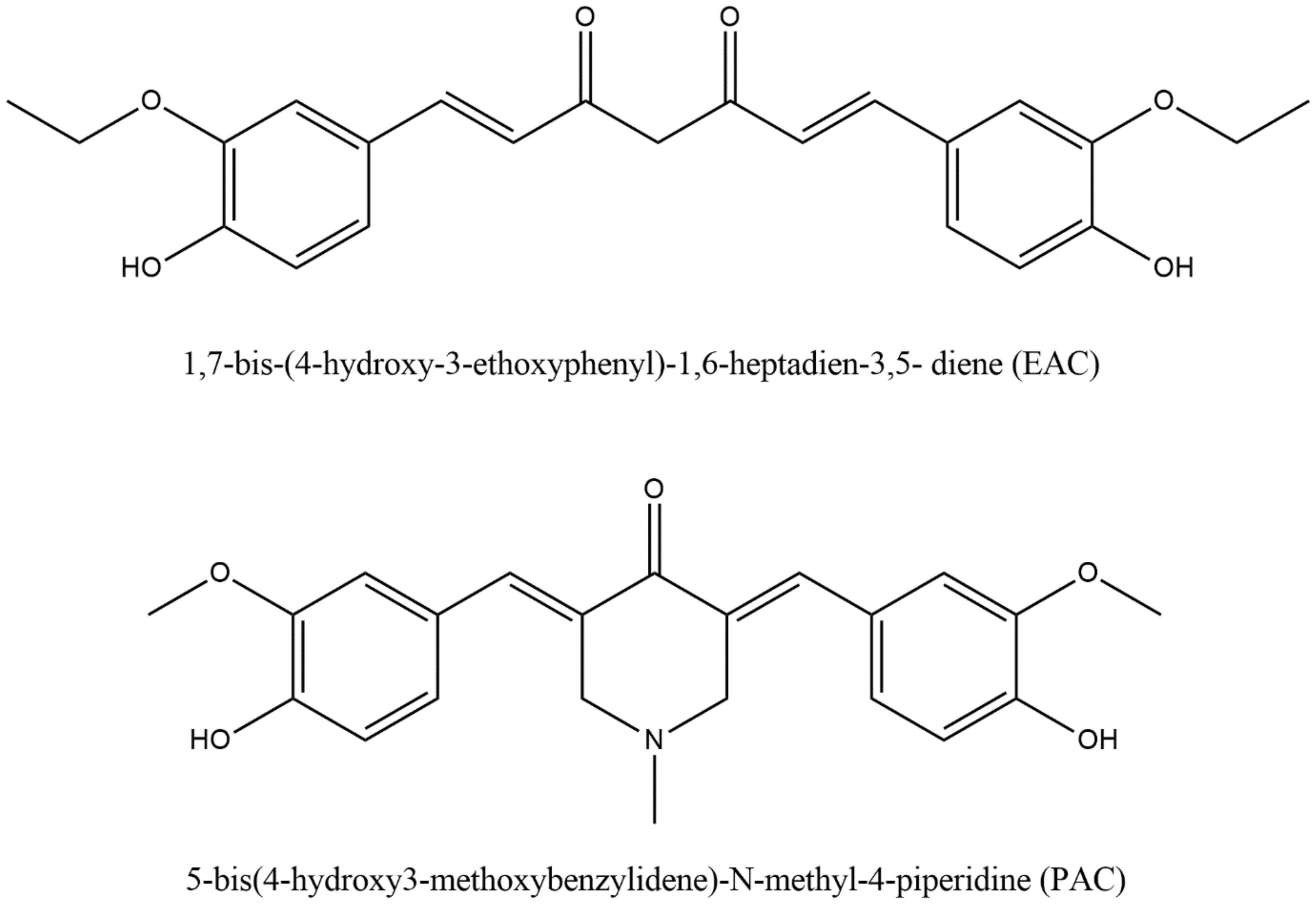
Curcumin analogs with strong anti‐breast cancer action.

Estrogen along with alpha and beta which are estrogen receptors has a substantial act on the development or proliferation of cancer in the breast. Among women, this type of cancer is said to be the most frequent one. Meanwhile, most of the persons diagnosed with cancers of the breast (2/3rd) have boosted their receptors related to estrogen, and this can be considered a vital activity to target these receptors in ongoing treatments focused on reducing such tumors (Herynk & Fuqua, 2007). A curcumin series in analogs have been created and investigated for the action of anti‐tumor properties. Significantly, also two non‐toxic analogs of curcumin have been studied resulting in suggestions of their anti‐breast cancer activities, which are 1,7‐bis‐(4‐hydroxy‐3‐ethoxyphenyl)‐1,6‐heptadien‐3,5‐diene (EAC) and 5‐bis(4‐hydroxy3‐methoxybenzylidene)‐N‐methyl‐4‐piperidine (PAC) (Figure 6). It has been indicated in the results that these mentioned analogs have comparatively greater blood stability than curcumin and greater bioavailability, biodistribution, and elevated water solubility. In addition, for starting apoptosis activity in breast tumors, they revealed effectiveness five folds higher than curcumin (Al‐Hujaily et al., 2011).

- Lung's cancer

**FIGURE 7:**
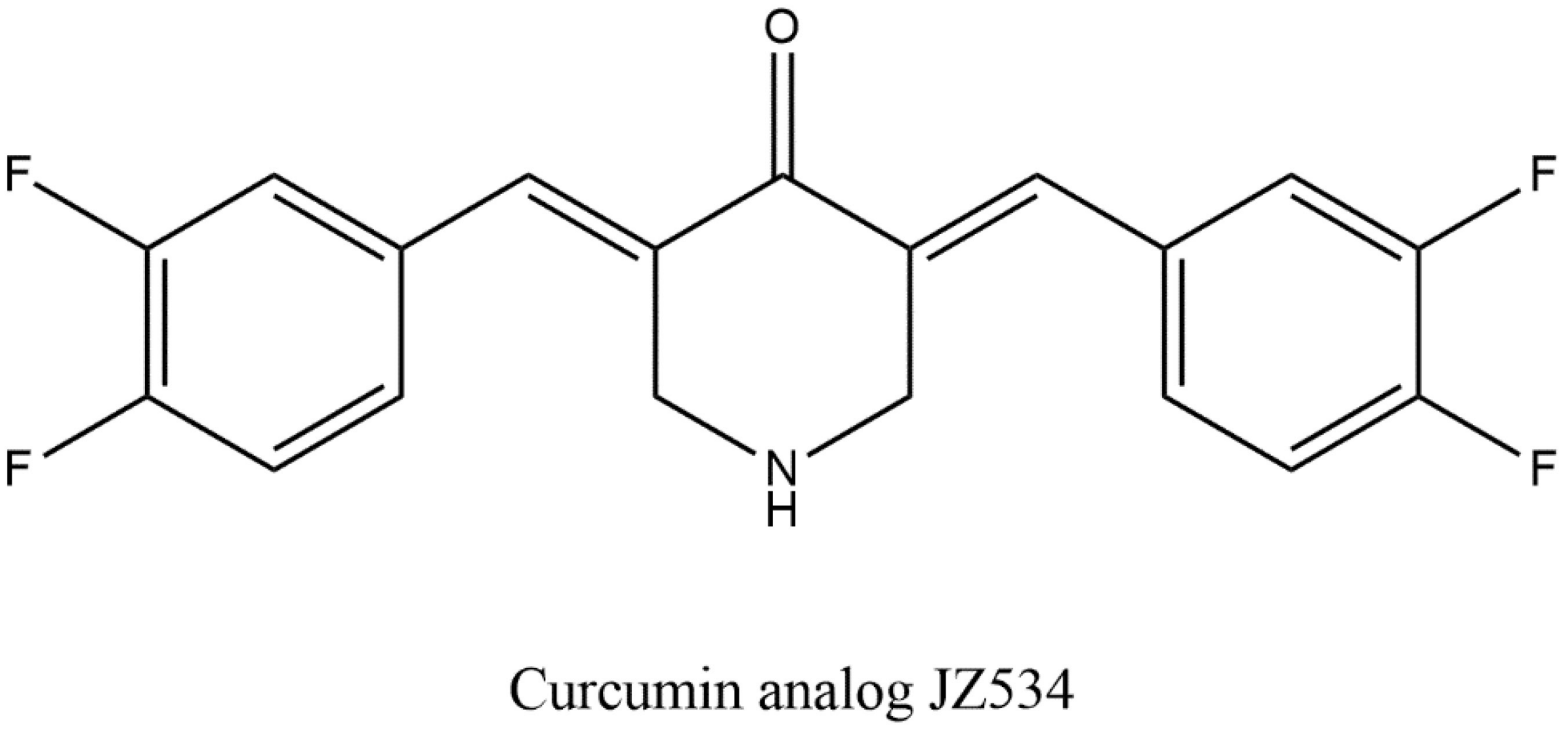
An analog of curcumin that fights lung cancer.

With a considerable higher rate of mortality and morbidity, cancer or tumors in lungs are considered in cancer types that are most fatal on global scale (Siegel et al., 2012). Nearly out of all persons affected with lung cancer, 85% were under the non‐small‐cell lung cancer (NSCLC) category. Also, two‐thirds of the NSCLC‐related cases were diagnosed at late stage which advanced and makes it quite complicated to treat this kind of tumor because of the insusceptibility to drugs (Detillon & Veen, 2018). A latest curcumin analog (JZ534) (Figure 7) has been created and studied on the lung cancer cell lines for its anti‐tumor activities. It exerted an imperative anti‐lung cancer influences through restraining the development, initiating apoptosis and regulating the processes of proteins related to apoptosis like caspase 3, Bax, and p53. Furthermore, JZ534 even at the same concentration indicated a greater anti‐tumor activity than that of curcumin (Wu et al., 2015).

- Prostatic cancer

**FIGURE 8:**
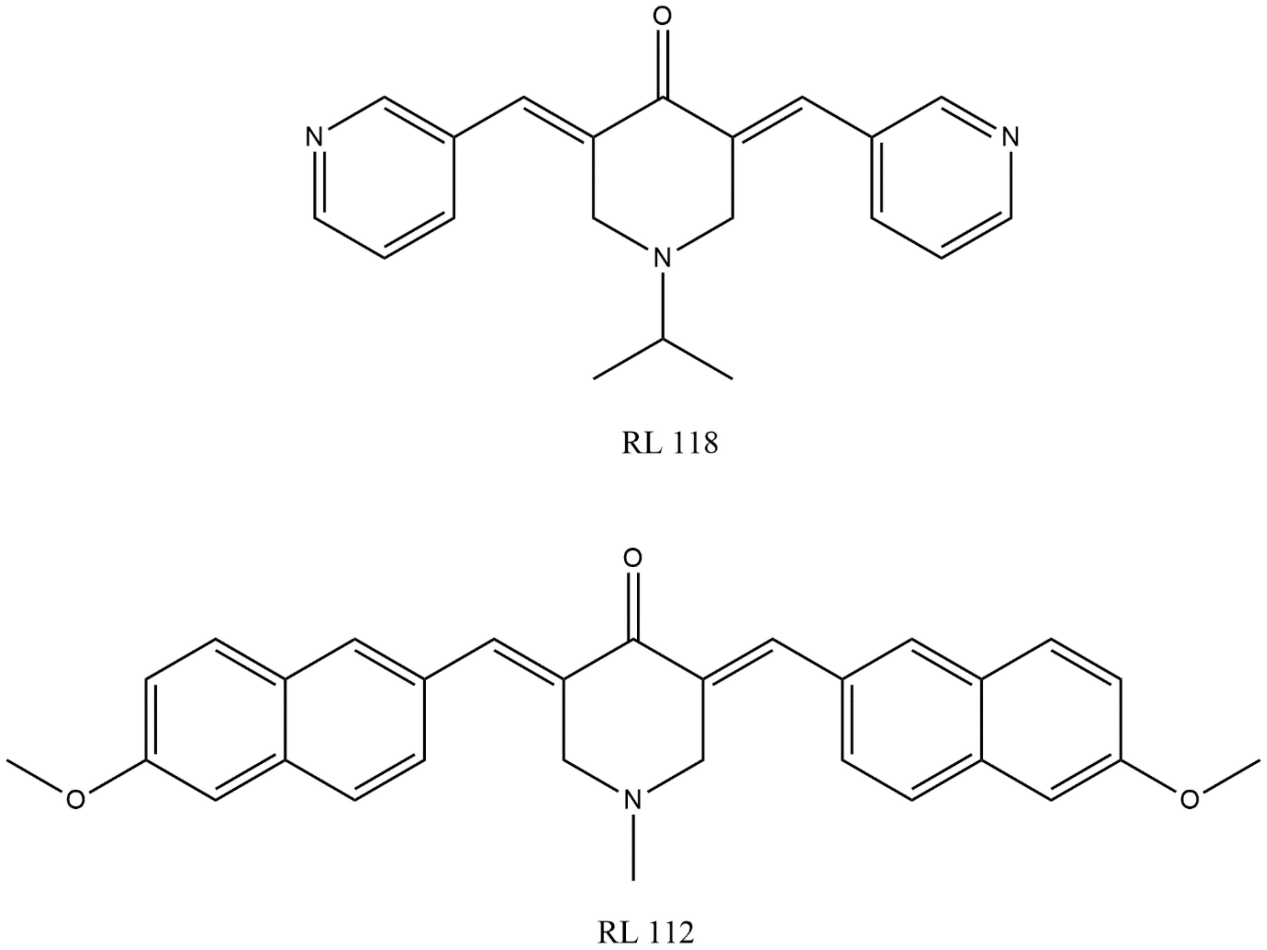
Activity of curcumin analogs versus prostatic cancer.

Prompt detection makes prostate cancer much more responsive to anti‐androgen therapy, but when the cancer cells start to resist to hormonal therapy, the cancer is classified as CRPC or castration‐resistant prostate cancer (Karantanos et al., 2013). It has been discovered that the RL121 and RL118 new analogs of curcumin (Figure 8) have specified influential cytotoxic effect by the result analysis on the DU145 and PC3 cells on CRPC. They revealed that these two analogs effectively upregulated the cell number in the phase of cell cycle called G2/M phase, caused programed cell death, and restrained the nuclear factor expression (NF)‐KB (Chen et al., 2018).

A latest clinical research performed on the patients having CRPC indicated that co‐administration of curcumin along with docetaxel in almost half of the patients produced a prostate‐specific antigen (PSA) response. In almost 88% of the patients in the preliminary 3 stages of medication cycle, PSA response activity was detected (Mahammedi et al., 2016).

- Pancreatic cancer

**FIGURE 9:**
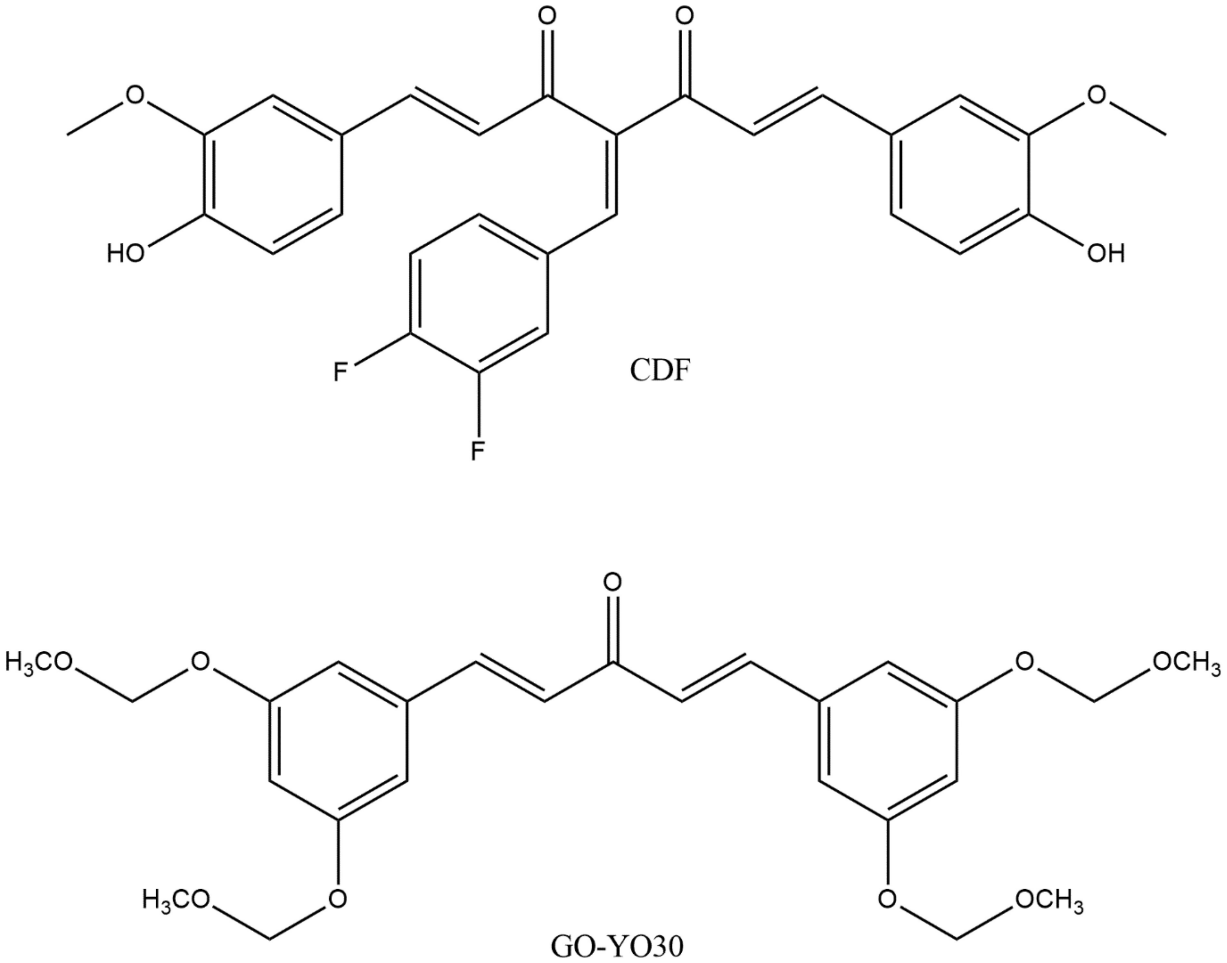
Compounds of turmeric that fight pancreatic cancer.

Pancreatic cancer is considered to have higher chances of malignancy‐relatable death worldwide. The percentage of deaths due to pancreatic cancer among all other cancer kinds is found to be approximately 7. The diagnosis and treatment of this type of cancer by using chemotherapy or radiotherapy have very less efficiency (Tang & Chen, 2014). GO‐Y030 (Figure 9) is also a curcumin derived, which has found to have more effective properties for stopping the pancreatic cell lines as compared to curcumin. This property of compressing the chances of survival of the titled cell lines can rely on the inhibition of STAT3 (Hutzen et al., 2009).

Because of the lacking results on radiation and chemotherapy as treatment techniques for this kind of cancer, different other kinds of techniques have been followed such as the use of Phyto‐related chemicals. The study results of an in vitro type study of CDF, termed as difluorinated curcumin, a derivative of curcumin (Figure 9), have revealed its properties to suppress the development and chances of survival of these cancer cells for treatment of several cancer cell lines related to pancreas (Bao et al., 2012).

- Brain cancer and glioblastoma

The chances of tumors in central nervous system (CNS) and brain cancer in the United Kingdom are anticipated to have a rise of 6% in the years 2014–2035 (Cancer Research UK, 2019). Glioblastoma (GBM), said to be most frequent kind of malignant nervous cancer in humans, claims nearly 15% of all CNS tumors (Klinger & Mittal, 2016). Curcumin has targeted certain different molecules, resulting in fighting brain tumors, can adopt various cellular pathways, which can be apoptosis, angiogenesis, invasion, autophagy, and metastasis. However, invading in the brain–blood barrier (BBB) is said to have the ability to decrease the anti‐cancer agent activities, and curcumin has the property to exceed the BBB in further stages (Perry et al., 2010). Furthermore, a report on in vivo studies involving human glioma cells (U‐87) xenotransplant in mice revealed a property in curcumin to compress angiogenesis of glioma via suppressing CD105 and CD31 mRNA endothelial cell markers and MMP‐9. In addition, curcumin showed the ability of increasing the levels of protein kinase 1 (DAPK1) seizing the G2/M cell cycle in U‐251 cells of malignant GBM, symbolizing the decrease in DAPK1 using curcumin initiate cell arrest and also activates caspase‐3 and seizes NF‐κB and STAT3 (Wu et al., 2013).

### 4.3 Anti‐diabetic activities

Diabetes is a metabolic issue that can be diagnosed as diabetes. This condition has allegedly been blamed for killing 1.5 million people in 2012, in addition to causing 2.2 million deaths from cardiovascular and other disorders (WHO, 2016). Indicated by some studies, diabetes has emerged as a burden mainly in less advanced countries internationally. Additionally, it has been anticipated that the frequency of diabetes would continue to rise globally, reaching 13.5% of the world's population in 2040 (IDF, 2015). The most frequent occurring is type‐2 diabetes mellitus (T2DM) in all the increasing number of diabetic patients. To tackle the increasing problem of diabetes worldwide, one requires efficient prevention and management techniques. T2DM is a disorder that can be prevented. Patients of pre‐diabetes or impaired glucose tolerance should also be considered for prevention, who are susceptible to T2DM. Two significant suggestions are lifestyle modification and pharmacological intervention (DeFronzo et al., 2015).

Curcumin was stated to consist of anti‐diabetic properties. The impact of anti‐diabetic property could be because by the anti‐oxidant potential of curcumin (Panchatcharam et al., 2006). Until today, many effective researches that firmly suggest the possible therapeutic impact of curcumin in the supervision of diabetes mellitus using its anti‐oxidative, anti‐inflammatory, hypoglycemic, and hypolipidemic effects (Alsamydai & Jaber, 2018). Curcumin can improve insulin effectiveness by interfering with other processes like by enhancing the liver glucokinase activity and resultantly improve the glucose homeostasis. Moreover, it has the ability to stimulate the activity of lipoprotein lipase for suppressing very low‐density lipoprotein and triglyceride resultantly decreasing the hypertriglyceridemia. On the third point, it can improve the level of glucose uptake extrahepatically through stimulation of GLUT4 (glucose transporter‐4) expression (Sharma et al., 2006).

Curcumin was tested and analyzed as a prevention medicine of type‐2 diabetes in pre‐diabetic patients by Aggarwal and Harikumar (2009). In comparison to the placebo group, the group that received it had a lower level of HOMA‐IR (insulin resistance index) and a relatively greater level of adiponectin. Results found that curcumin introduction can have positive impact to a pre‐diabetic human population. Kim and his colleagues performed experiments and reported that decreasing levels of glucose effects of curcumin could be because of falling levels of hepatic glucose output via gluconeogenesis reduction (Farhangkhoee et al., 2006). Curcumin has also been shown to lessen the severity of diabetic micro‐ and macrovascular issues, such as retinopathy, diabetic nephropathy, and cardiomyopathy (Edward et al., 2019; Ran et al., 2019).

An organized research on RCTs, called as random control trials, was performed. Curcumin considerably suppressed glycosylated hemoglobin (HbA1c) in pre‐diabetic population. In addition, T2DM group people were observed with substantial decrease in both fasting plasma glucose and HbA1c. Capability of lipid profile betterment was also indicated (Poolsup et al., 2019). One more study reported that introduction of Wistar, diabetic and alloxan‐induced rats for 2 h with compounds 10 and 4, which are analogs of curcumin, gave results which indicated low blood glucose level, just like levels obtained by anti‐diabetic drug glipizide administration (Das et al., 2016). In studies reported by Lu et al. (2014), db/db mice were introduced with a curcumin dose of 200 mg per kg per day for a time period of 18 weeks, and results indicated having a considerable reduction in levels of glucose in blood, body mass, and albumin in urine. Likewise, Shao et al. reported in Shao et al., 2012 that the C57BL/6J mice fed with a high‐fat diet with 0.4 percent curcumin for 28 weeks, with 2 days of injection each week, showed reduced rates of obesity and insulin resistance. Furthermore, there was an observation of significantly reduced levels of mass of fat in epididymal cells and blood glucose, while serum adiponectin amount was boosted.

There have been clinical trials done on humans with a dose amount of 200–500 mg everyday of curcumin for about 12 weeks. It is revealed in many studies that by employing various systems for drug delivery—such as nanoparticles loaded with curcumin, inclusions of cyclodextrin, microemulsions, and liposomes—an improved mode of action and enhanced curcumin bioavailability have been indicated (Flora et al., 2013; Hewlings & Kalman, 2017). The natural possessions of curcumin administration to diabetic and healthy patients are briefly tabulated in Table 2.

**TABLE 2:** Anti‐diabetic effects on human studies.

Ailment	Application amount	Interval	Serum properties	References
Strong individual	6.1 g	About 35–60 min	Raised level of insulin	Wickenberg et al. (2010)
T2DM sufferers	199.90 mg/day	98 days	Decreased levels of blood sugar and HbA1c	Sukandar et al. (2014)
T2DM sufferers	80.2 mg/day	91 days	Decreased levels of fasting triglycerides, LDL, and glucose	Rahimi et al. (2016)
Patients with pre‐diabetes	1500.1 mg/day	273 days	Lower values of diabetes, insulin, and glucose	Chuengsamarn et al. (2012)
Obese diabetic individuals	300.02 mg/day	91 days	Decreased levels of A‐FABP, TNF‐α, and CRP	Na et al. (2014)
Those with impaired fasting glucose levels	125.12 mg/twice/day	56 days	Lower levels of insulin fasting and triglycerides but increased levels of HDL	Cicero et al. (2017)

### 4.4 Anti‐obesity activities

Obesity is considered a health crisis on global level, and the concern regarding this field of line is increasing gradually majorly because of the impacts it had on death rate, economic status, and morbidity worldwide (James, 2004). Due to its great risks leading to severe diseases, obesity is considered as a health concern consisting of diabetes mellitus, osteoarthritis (OA), heart disease and stroke, and hypertension, etc. Obesity is specified by having metabolic inflammation of short grade but still can be upregulated if preadipocyte growth is achieved. Therefore, dietary supplements having anti‐inflammatory actions, like curcumin, are said to be therapeutically important because of their properties of enhancing preadipocyte proliferation and cellular oxidation (Bradford, 2013). Curcumin comes under the spice category of food and other nutraceuticals that are now known for their prevention properties against the metabolic disorders related to obesity and its chronic impacts (Aggarwal, 2010). Curcumin has major impacts of suppressing macrophage infiltration on white adipose tissue (WAT) to control the production of provocative adipokine and to stimulate adiponectin adipocyte synthesis (Bradford, 2013).

The base for further studies and research on how obesity affected persons was provided by results in which curcumin showed an imperative effect on lipid status of body as also fat content on patients who were tested. Curcumin impacts on obesity were indicated by different clinical trials. The primary one studied the effect of curcumin supplementation on some lipid‐related parameters like levels of glucose in obesity patients and body‐mass index (BMI) using the oral path. The results obtained proved that there were imperative variations but only in the levels of triacylglycerol (TAG); meanwhile, all other parameters remained the same when curcumin was administered for 30 days (Sahebkar et al., 2013). The decrease in body‐weight and basal metabolic rate were also observed in research who analyzed these parameters in non‐alcoholic fatty liver disease (NAFLD) patients (Rahmani et al., 2016). The outcomes directed that the case, where turmeric was administered for almost 4 weeks with a dose amount of 2.8 per day in female patients of total inflammation obesity, did not produce a variation in metabolic condition, and also there was no change in the parameters related to inflammation or oxidative stress (Nieman et al., 2012). Although the results reported by above studies were not encouraging at all, still in recent findings, a positive impact of curcumin is shown on the BMI and body weight. After the curcumin analysis of about 1.6 g per day for almost 1 month given via the oral route (mixed with 8 mg piperine), observations indicated significant improvements in body fat, weight, and parameters related to them, and also the basal metabolic index and body weight were reported to be decreased by the person who conducted the experiments in subjects of NSFLD (Di Pierro et al., 2015; Rahmani et al., 2016).

Curcumin can decrease preadipocyte differentiation, resulting in the reduction of the adipocyte concentration and fat contents of adipose tissues (Ahn et al., 2010). In conducted experiments with the mixture of insulin along with dexamethasone and isobutylmethylxanthine (IBMX), results indicated the improvement in development and proliferation of murine 3T3‐L1 preadipocytes and primary preadipocytes in human body (Zhao et al., 2011). The studies with administration of curcumin with 5–20 mM reported the creation of a suppression of adipocyte development that was dose dependent as indicated by the analysis of specific gene expression of adipocyte (PPARc) termed as peroxisome proliferator‐activated receptor *c*, resistin, adiponectin, and cytosine‐cytosine‐adenosine‐adenosine‐thymidine (CCAAT) enhancer protein which attaches a specific protein (C/EBPb) leptin (Kim et al., 2011).

Obesity can have a significant impact on epigenetics as a result of harmful environmental exposures that alter the DNA directly and increase vulnerability to obesity‐related disorders such various cardiovascular diseases (CVDs) and type 2 diabetes (Slomko et al., 2012). Although curcumin was mainly studied and experimented for the predictive task against cancer, it can also accelerate modulations in epigenetic system that directly targets obesity leading genes. Techniques that can be used for this purpose include global hypomethylation of DNA, the suppression of enzyme histone acetyl transferase p300/CBP, micro‐RNA processes, and histone modifications (Fu & Kurzrock, 2010). Many experiments and studies have been conducted on curcumin for its properties affecting mainly the CpGs demethylation in gene promoters in DNA hypomethylation. Curcumin is known to have the property of returning the Nrf2 expression through the activities occurring because of DNA hypomethylation and, subsequently, acts to create anti‐oxidative actions that are Nrf2‐mediated and also boost up the pathways of cellular defense against stress (Khor et al., 2011).

### 4.5 Cardioprotective effects

Heart‐related illness, mostly known as cardiovascular diseases, has become a major health concern worldwide as they are the reason of most of the deaths globally, and the mortality and morbidity are still increasing. It is estimated that in America, almost 40.5% people will be suffering from CVDs by the year of 2030. This may lead to about 276 billion dollars in secondary costs and 818 billion dollars for medical costs (Heidenreich et al., 2011). It is also known that most significant cause of CVDs is obesity. Other chief reasons of CVDs are characterized in two categories: one is non‐traditional (novel) and other is traditional. Hypertension, ischemic heart disease, diabetes, and consumption of alcohol are known to be the major causes of heart failure (Kemp & Conte, 2012; Pontes et al., 2010). Traditional risk factors of CVDs comprise smoking cigarette, hypertension, diabetes mellitus, hypercholesterolemia, obesity, and high blood pressure, which are often modifiable. One of the fundamental risk factors (traditional) for CVDs is obesity (Balagopal et al., 2011; Roger et al., 2011).

Curcumin has oppressive properties on heart failure as well as in cardiac hypertrophy and is a renowned inhibitor of p300 (Chowdhury et al., 2013). GATA4 has been proved as a crucial transcription aspect for heart development and pathology. Several studies were done for additional reveals of its potential mechanisms, which exposed the perilous contribution of GATA requisite protein 4. Curcumin not just reserved the collaboration between GATA4 and p300 but also repressed the localization of GATA4 inside the nucleus, which leads toward the reduction acetylation of GATA4. This is the reason of its protecting outcome against hypertrophy of heart (Ahuja et al., 2011; Thompson et al., 2015).

**FIGURE 10:**
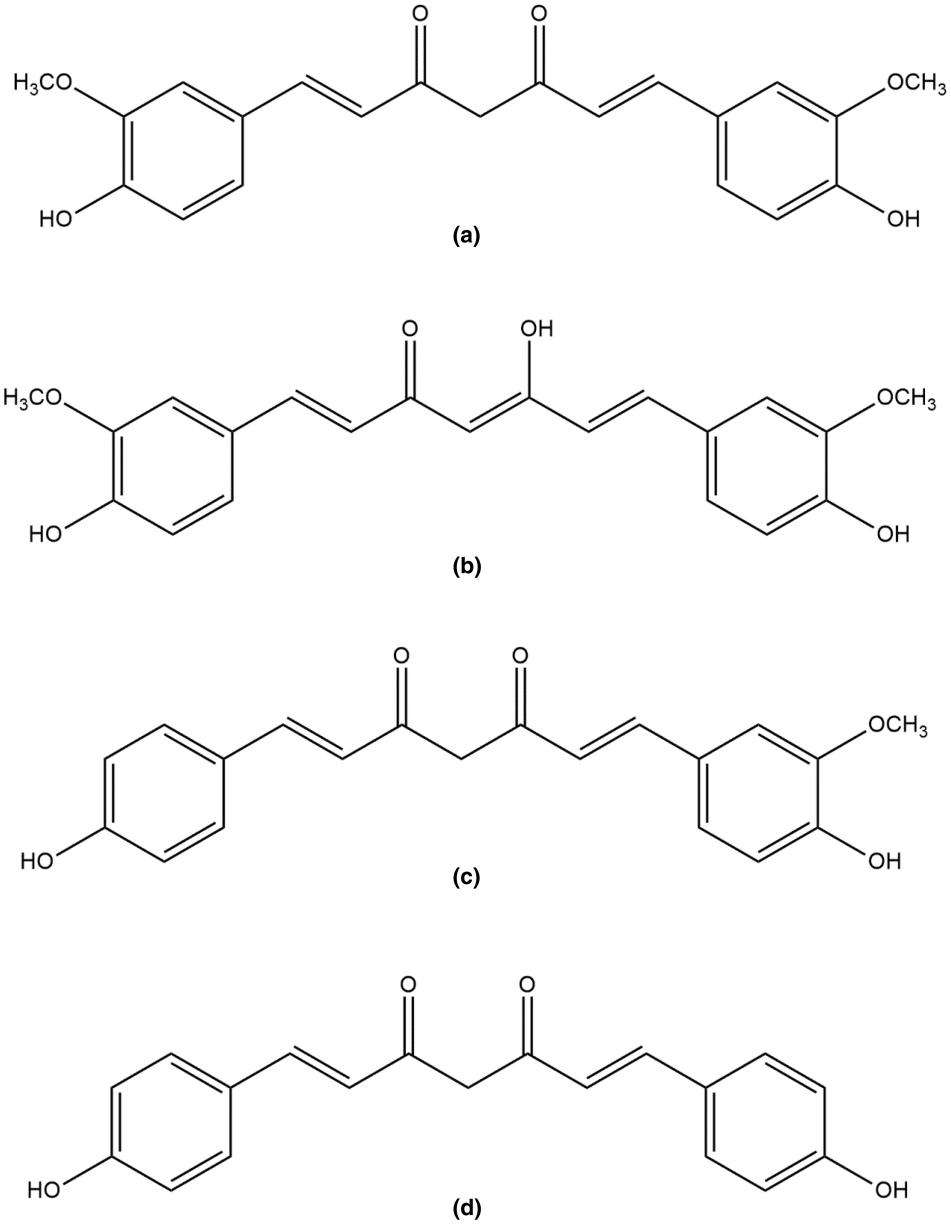
Curcumin derivatives in treatment of CVDs.

Both synthetic and natural (Figure 10) curcumin derivatives have already been assessed for their useful properties in the treatment or inhibition of CVDs. Anti‐coagulative actions of curcumin and its derivative (BDMC) were assessed by Kim et al. (2012) by evaluation of the partial thromboplastin time as well as prothrombin time (aPTT and PT, respectively) and the production of thrombin and activated factor *X*. Treatment with curcumin and its derivative (BDMC) abridged the generation of thrombin, extended PT and aPTT, and triggered factor *X*, showing effective anti‐coagulant potential. It was also revealed in this study that curcumin has more anti‐coagulant potential than BDMC. Li, Li, et al. (2015) and Li, Wu, et al. (2015) created the prenylated and allylated mono‐carbonyl derivatives and assessed their defensive actions against myocardial ischemia reperfusion injury.

Several researches on many animal models have investigated the protecting properties of curcumin on empirically persuaded cardio toxicity and hepatotoxicity, with diverse biochemical constraints like anti‐oxidants in marked tissues and serum marker enzymes. It was also found that curcumin treatment reduced the augmented comparative weight of heart and liver in isoproterenol persuaded heart necrosis and CCl4 tempted liver damage (Alsamydai & Jaber, 2018). Curcumin has been shown to have cardioprotective effects in an in vivo investigation on rats with myocardial ischemia due to its capacity to reduce free radical production and improve enzyme detoxification activities (GSH S‐transferase) (Miriyala et al., 2007). Another study was done on an animal model by Chakraborty et al. (2017), which showed that amalgamation of piperine and curcumin can enhance serum levels of anti‐oxidants and lipids as well as histopathological and electrocardiographic findings. It was also testified by Bhullar et al. (2013) that curcumin and its derivatives have resilient ability to protect from cardiovascular disease by inhibiting angiotensin‐converting enzyme (ACE) at 10 μM.

### 4.6 Anti‐inflammatory role

Ionized radiations in contact to normal cells can cause inflammation which can have some side effects. A common side effect of radiotherapy is chronic inflammation, and it was observed that it can obstruct repair pathways of DNA and encourage genomic volatility over incentive of free‐radical creation (Najafi et al., 2018). The skin's dermatitis, fibrosis and pneumonitis in the lung, enteritis in gastrointestinal track (GIT), edema in muscles, and proctitis in intestine are the results of side effects of chronic inflammation that appear in different organs (Yahyapour et al., 2018). Dendritic, macrophages, and lymphocytes cells are those inflammatory cells that have a substantial role in retort of cancer and its therapy (Jain, 2013). It was found that these cells can destroy cancer cells, but latest studies showed that provocative responses by these cells have a part in angiogenesis and tumor growth (Kershaw et al., 2013).

Many chronic diseases happen due to the oxidative stress in such a way that one disease can be a pathway toward a next disease in cascade. The relationship between oxidative stress and inflammation was demonstrated by Biswas (2016), which revealed that oxidative stress is led by the inflammatory cells which, in turn, release many reactive species at inflammation location. Traditionally, treatment of some inflammatory ailments can be done with the help of curcumin (Aggarwal et al., 2007). A study found that curcumin has the capacity to inhibit the ability of some transcription factors, primarily NF‐B, to promote inflammation (Shehzad et al., 2013). Studies also revealed that lipoxygenases and prostaglandins are involved in the fabrication of free radicals, and they promote inflammatory signs, and this metabolism is affected by curcumin (Aggarwal & Harikumar, 2009).

Anti‐arthritic potential of curcumin is confirmed during many experimental trials done on patients suffering from rheumatoid arthritis (RA) and osteoarthritis (OA), which showed anti‐inflammatory worth of curcumin (Belcaro et al., 2010; Dcodhar et al., 2013; Kertia et al., 2012). After using curcumin supplementation, a noteworthy drop in blood cytokine (MCP‐1, TNF‐a, TGF‐b, and IL‐6) levels was noted in patients with metabolic diseases (Panahi et al., 2015). Hence, all these outcomes strongly backing the declaration that application of curcumin can be helpful in the treatment of several inflammatory diseases.

**FIGURE 11:**
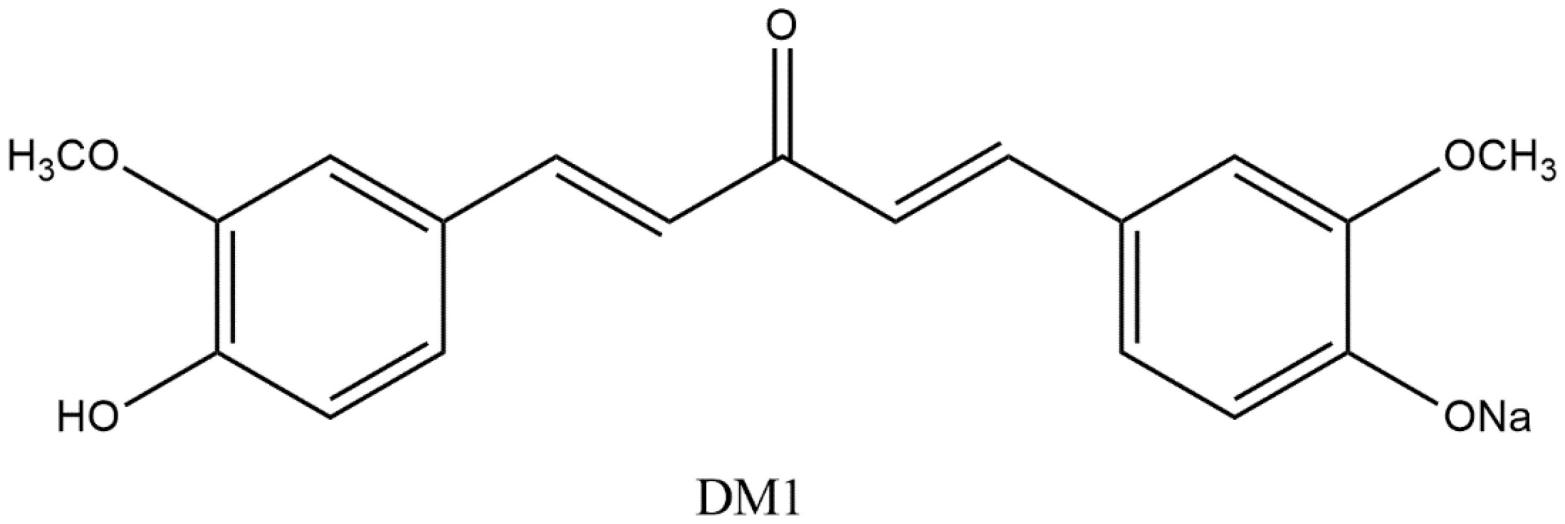
An analog of curcumin with strong anti‐inflammatory properties.

Scientists have prepared an analog of curcumin known as DM1 (showed in Figure 11) and assessed its result on the inflammatory mediators, which proved that DM1 has potential to overwhelm the hindering cyclooxygenase‐2 (COX2) and inducible nitric oxide synthase (iNOS) (Kim et al., 2009).

An intracellular signaling cascade that is started by some active nitrogen/oxygen species can enhance the production of pro‐inflammatory genes. Inflammation is the root cause of many chronic disorders (Jurenka, 2009; Recio et al., 2012). Parkinson's disease, epilepsy, Alzheimer's disease (AD), acquired immunodeficiency syndrome (AIDS), multiple sclerosis, cerebral injury, metabolic syndrome, cardiovascular disease, bronchitis, colitis, allergy, cancer, asthma, renal ischemia, arthritis, psoriasis, obesity, diabetes, fatigue, and depression are just a few of the illnesses that inflammation can cause (Panahi et al., 2016). Curcumin has shown noteworthy anti‐inflammatory activities in chronic as well as in acute replicas of inflammation. It was found to be only half as effective in chronic tests but in the carrageenan edema test as powerful as phenylbutazone (Naik et al., 2011). Many transcription aspects, protein kinases, cytokines, enzymes, adhesive molecules, and redox status have been associated to inflammation, and curcumin has been shown to regulate them (Kim et al., 2010). A study was done on 6 persons to check the safety of curcumin which confirmed anti‐inflammatory potential of it. This study also demonstrated that curcumin has the ability to constrain an amount of dissimilar molecules that has a role in inflammation (Hilles & Mahmood, 2016).

During investigational researches, some experimental trials showed that tumor therapy has adverse effects, like inflammatory conditions, and curcumin has the potential to protect from these side effects. Examples of such side effects of cancer therapy are mucositis and dermatitis, which can be retorted by curcumin. The utmost common side effect of treatment (radiography), which is used for different kinds of cancers, is dermatitis. After radiotherapy of breast cancer, it is assessed that 95% patients suffer from dermatitis. Protective effect of curcumin was found in a cream which was prepared comprising sandal wood oil and curcumin by Palatty et al. (2014) for the patients suffering from neck and head cancer who undergo radiotherapy. The initial trials displayed good outcomes. These results exposed that this cream has ability to assuage dermatitis (Grade 3) on patients undergone through radiotherapy. Another study showed that utilization of this cream exhibited reduction of dermatitis signs during 2nd and 3rd weeks. During this study, patients were exposed to 2 Gay per day, five times in a week of radiations (total dose 50 Gay) for five successive weeks (Rao et al., 2017). Favorable influence of curcumin was also found on 30 patients with chest cancer by Ryan et al. (2013) on radiation‐induced dermatitis in a double‐blinded experimental test study. After the 5th week, drop in dermatitis was detected.

### 4.7 Anti‐asthma

Asthma is a chronic inflammatory disease, which is known to be among the enormously widespread diseases of world. Airway remodeling, capricious restriction in air‐flow, and tracheal hyper‐responsiveness are core features of asthma, whereas coughing, wheezing, and hyperventilation are the main symptoms (Global Initiative for Asthma, 2016). Pervasiveness of asthma is growing for the previous 20 years, and it is leading toward sickness of majority of people globally. In children, contact to corticosteroids for longer period of time can have severe adverse effects. Main aims of asthma treatment are to retain the symptoms at lowest level, long‐term control of inflammation, and amending function of lungs. Hence, novel, effective, and harmless medicines are required for asthma treatment.

Research conducted by Manarin et al. (2019) exposed that *Curcuma longa* roots powder, when given to the asthma patients (adults and children), led to better disease control, fewer awakenings during night sleep, and reduced recurrent use of SAβAA, after third and sixth months, as compared to the standard treatment. Curcumin has found to be alleviating rhinorrhea, sneezing, and nasal congestion by decreasing the resistance in nose airflow. In research conducted on asthma affected rats, curcumin was directed over nasal way which upheld structural integrity and repressed allergic airway inflammations. Curcumin administration at different proportions evidently controlled airway obstruction and inflammation in rat study by hindering COX‐2 expression and PGD2 release in ovalbumin (OVA) primarily through controlling sPLA2 activity and levels of cytokine (TNF‐α, IFN‐γ, and IL‐4, 5). Moreover, in asthma progression rats, curcumin also caused repression in activation of JNK54/56, ERK 42/44, and p38 mitogen‐activated protein kinase (MAPK) (Subhashini et al., 2013). In both chronic and acute asthmatic animal models, evidences have shown that intranasal curcumin averts obstruction, remodeling and airway inflammation by prostaglandin D2 release and regulating instigation of mitogen‐activated protein kinase (JNK, p38, and Erk) (Chauhan et al., 2014; Subhashini et al., 2013).

According to Mason et al. (2015), airway eosinophilia, goblet cell hyperplasia, hyperresponsiveness to exogenic and endogenic allergens, and mucus hypersecretion are also indications of asthma. It is also found that curcumin helps in the management of allergic asthma. This anti‐asthmatic influence of curcumin is credited due to the raise in aquaporin expression levels and reduction of pro‐inflammatory cytokines (Shahid et al., 2019). Eosinophiles (a variety of white blood cells) produce free radicals and inflammatory mediators which play a vigorous part in pathogenesis of asthma. There is a group of cytokines, known as interleukins, that intricate in the initiation procedure of eosinophils, for example, IL‐4 and IL‐5 (Pulido‐Moran et al., 2016). It is considered that controlling their activities can be a new approach against asthma. Additionally, intranasal curcumin has anti‐inflammatory properties, which are useful in treating this type of allergic asthma (Chauhan et al., 2017).

In asthmatic rats, protective effects of curcumin have been shown on lungs compulsive features and tracheal receptiveness (Shakeri et al., 2018). During this research, a significant reduction in tracheal sensitivity to OVA and methacholine as well as pathological features like emphysema, interstitial inflammation, bleeding, and interstitial fibrosis was detected in curcumin‐treated groups. Research was directed by Park et al. (2020) using a rodent asthma model, whose aim was to assess anti‐asthmatic properties of Cur‐PLGA‐DPPs [curcumin‐containing poly (lactic‐*co*‐glycolic acid)‐based microscale discoidal polymeric particles]. Treatment with curcumin‐containing poly (lactic‐*co*‐glycolic acid)‐based microscale discoidal polymeric particles showed better therapeutic efficacy, at same curcumin concentrations, than treatment with free curcumin. These outcomes proposed that the curcumin‐containing poly (lactic‐*co*‐glycolic acid)‐based microscale discoidal polymeric particles can be possibly used as a lung‐targeted asthma treatment. Likewise, it was also demonstrated that liposomal curcumin repressed the imperative pro‐inflammatory indicators intricated in the pathogenesis of asthma, which could be an auspicious asthma cure intervention (Ng et al., 2018). Babaei et al. (2020) suggest utilizing curcumin as a potential treatment for COVID‐19. Further research studies are required on this subject.

### 4.8 Neuroprotective role

According to World Health Organization (WHO), neurological ailments are well‐defined as various pathologies of the peripheral and central neuronal system, such as the peripheral nerves, brain, spinal cord, cranial nerves, nerve roots, autonomic nervous system, muscles, and neuromuscular junction. Traumatic disorders of the nervous system, epilepsy, Parkinson's disease, Alzheimer disease, brain tumors, and migraine are produced by above‐mentioned disorders (Pulido‐Moran et al., 2016). Curcumin interacts with and influences a number of molecular targets, including the anti‐oxidant system, growth aspects, kinases, and inflammatory cytokines. These activities of curcumin demonstrate its function as a neuroprotectant.

Neuroprotective influence of curcumin was proved by Li, Li, et al. (2015) and Li, Wu, et al. (2015). According to this study, curcumin has the ability to regulate AMPK (adenosine monophosphate–activated protein kinase) activity, which decrease ROS‐related endoplasmic reticulum stress that is linked to neuronal harm. Parallel findings were observed by Fu et al. (2016), who conducted their study to check the protective effect of curcumin on PC12 cells in H_2_O_2_‐induced neurotoxicity. Studies done by above‐mentioned researchers proved dysregulation in AKT and MAPK (mitogen‐activated protein kinase) pathways by curcumin, which reduced apoptotic cells through inhibition of ROS accumulation. It is also proven that curcumin executes anti‐inflammation effects through p38, MAPK, and TLR4 signaling and neuroprotective effects through mTOR, PI3K, and Akt signaling (Huang et al., 2018).

The utmost common reason of dementia (forgetfulness) worldwide is Alzheimer disease. This disease is advanced and chronic neurodegenerative syndrome of the brain, which is clinically categorized by the worsening in the crucial signs of communicative and intellectual abilities. Researchers like Ghosh and Gomes (2016) have described curcuminoids as anti‐Alzheimer agents. Since AD has mechanisms with complex pathology and multifactorial etiology, there is a great requirement for searching the therapeutic agents with pleiotropic actions aiming some pretentious procedures (Chen et al., 2011). There are several compounds that achieve these possessions and, among them, curcumin displayed a powerful anti‐amyloid‐β possessions with significant anti‐oxidant as well as anti‐inflammatory activities (Sanjeeva et al., 2015).

**FIGURE 12:**
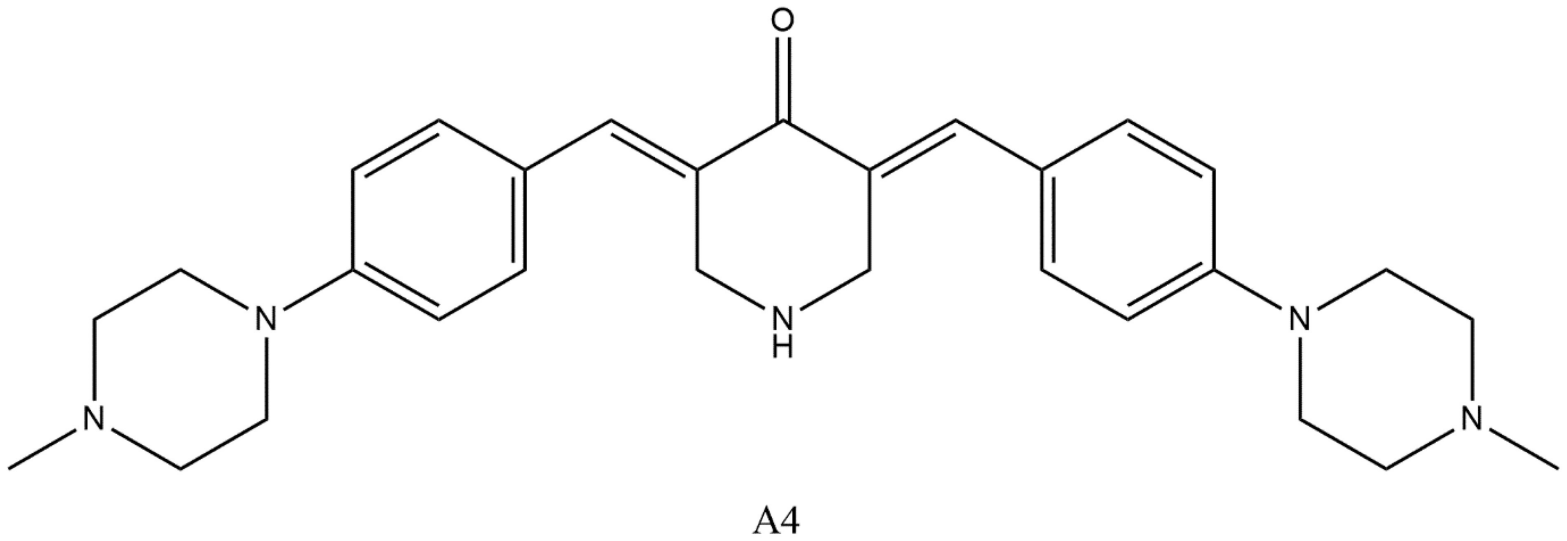
Curcumin derivative with anti‐Alzheimer activity.

Chen and his team produced a sequence of curcumin by‐products and, then, evaluated their worth in treatment of AD. These derivatives displayed higher repressive activity against Aβ accretion as compared to curcumin. Additional inquiries strengthened the fact that a derivative of curcumin (Figure 12) termed as A4 exhibited improved outcomes as compared to other used derivatives of curcumin. The conclusions of these studies gave rise to an idea to additional optimization of the structure of A4 to increase the efficiency of multifunctional anti‐Alzheimer agents (Chen et al., 2011). During recent studies, comparatively lesser dosages (80–180 mg per day) of new curcumin formulations were used, which showed a higher bioavailability. This experiment verified that both chronic and acute activities enhanced working memory tasks as well as sustained attention instantly after using first dose, whereas after the fourth week of usage, it improved contentedness memory, alertness, and mood (Cox et al., 2015; Small et al., 2018).

Another neurodegenerative ailment is Parkinson's disease, which is triggered in basal ganglia by slow injury to dopaminergic neurons, selectively in the substantia nigra (SN) pars compacta area (Mandal et al., 2020). A noteworthy reduction of the thiol anti‐oxidant GSH in these nerve cells is an imperative biochemical feature of pre‐symptomatic Parkinson's disease, which results in dysfunction of mitochondria, oxidative stress, and cell death. Curcumin supports the treatment of PD by protecting against protein oxidation, by reinstating repletion of GSH levels, and by preserving complex I activity of mitochondrial (Perry et al., 2010). Parkinson's disease and various synucleinopathies are known to be associated with irregular and overexpressed accumulation of mixed synuclein (S). Although synuclein (S) can adopt and misfold a variety of geometries, most recent studies suggest that oligomeric forms are the most lethal species. Curcumin has the ability to decrease intracellular ROS levels, ease in αS‐induced harmfulness, and defend cells against apoptosis (Li et al., 2014). Hence, it is evidenced that curcumin can be employed as an anti‐Parkinson agent.

### 4.9 Hepatoprotective effects

One the utmost important organ in our body is the liver that accomplishes several physiological utilities. The chief roles of liver are storage, secretion, and metabolism. Another role of liver is to detoxify exogenic substances and endogenic waste products (Khan et al., 2019). Liver is known to be the utmost crucial site for metabolism of endogenous and exogenous substances. Any ailment of liver can disturb hepatic biochemical and physiological functions (Madrigal‐Santillán et al., 2014). Alcoholic liver disease (ALD), hepatitis C, hepatitis B, liver cirrhosis, hepatocellular carcinoma (HCC), non‐alcoholic fatty liver disease, and hepatic failure are included in those disorders (Wang et al., 2014). It was revealed in a survey conducted in Pakistan that males are more affected by HCC than females, and its frequency was observed to be 2.8/100,000 for females and 7.6 for males (Hafeez Bhatti et al., 2016).

The primary research evaluating curcumin hepatoprotective potential was done on people suffering from tuberculosis, in which the focus was concerned on their capability to avert anti‐tuberculosis treatment (ATT)‐induced hepatotoxicity. A notable reduction in erythrocyte sedimentation rate and gain in weight were detected in the treated people (Adhvaryu et al., 2008). Rahmani et al. (2016) conducted a study in which people suffering from NAFLD were given supplementation of curcumin. A substantial drop in body weight, ALT and AST levels, and BMI with an enhancement in lipid profile status was observed at same time, which amended hepatic ultrasonographic results in the patients. Kim et al. (2013) also conducted a study to check hepatoprotective potential of curcumin, where turmeric powder (fermented) was used to assess its effects on ALT levels. AST, total bilirubin (TB) levels, lipid profiles, and gamma‐glutamyl transferase (GGT) were also measured in this research. It was observed that ALT and AST levels were considerably reduced in the group treated with fermented turmeric powder after 12 weeks of treatment. Considering other constraints, lipid profiles, serum alkaline phosphatase (ALP), and TB levels were not changed, while GGT levels exhibited an inclination to decline. Curcumin also exhibited a promising potential against non‐alcoholic fatty liver disease, which is a long‐lasting liver disorder categorized by neutral lipids gathering in liver cells.

Anti‐oxidant and anti‐inflammatory influence as well as anti‐fibrogenic potential of curcumin possess beneficial effects during hepatic ailments. Additional studies also revealed that curcumin raised SOD and GSH levels and reduced TBARS (thiobarbituric acid reactive substances) level in the liver homogenates when supplemented in LPS‐challenged mice (Ghosh et al., 2011). A study conducted by Abdel‐Daim and Abdou (2015) also proved that curcumin has the ability to enhance the hepatic total anti‐oxidant capacity and the xenobiotic detoxifying enzymes activity but lowering the lipid peroxidation, which results in reduction of iron‐induced liver damage. Moreover, curcumin can up standardize the cytoprotective enzyme HO‐1, inhibiting the ROS development in hepatic cells (Černý et al., 2011; Dai et al., 2015). It was also verified by Wang (2015) in a research study that curcumin improved the mitochondrial anti‐oxidants and decreased the aminotransferase and NASH action in animals with high‐fat‐induced NASH. Additionally, curcumin can also lower TNF‐α level, decrease mitochondrial ROS, and improve mitochondrial function.

Tung et al. (2017) performed a research study with the objective to formulate and determine physicochemical features of phytosome curcumin. Assessment of hepatoprotective potential of phytosome curcumin with comparison of free curcumin was done on paracetamol‐induced rats. This research revealed that phytosome curcumin had a robust protecting consequence in contradiction of paracetamol‐induced severe hepatic damage in rats. Phytosome curcumin possess hepatoprotective effect in rats by reducing liver enzyme and lipid peroxidation and by enhancing levels of anti‐oxidant enzymes on paracetamol‐induced damage.

### 4.10 Anti‐hypertensive potential

Another significant medical syndrome is hypertension, which is affecting around ¼ population worldwide, and it is estimated that it will raise to 29% by 2025 (Mittal & Singh, 2010). Estimations indicated that around one billion people are suffering from hypertension worldwide (Patel et al., 2017). Another study showed that there will be around 1.56 billion individuals suffering from hypertension in 2025 (Balakumar et al., 2016). HTN or chronic hypertension can cause a variety of medical issues, such as myocardial infarction, cardiovascular diseases, stroke, renal failure, and retinopathy (Anderson & Morrow, 2017; Bavishi et al., 2017; Ettehad et al., 2016; Jordan & Powers, 2016).

HTN has become a great financial load on humanity and the foremost root of sickness as it is connected with the risk of many chronic diseases. There could be some adverse health effects of using anti‐hypertensive medicines, and its efficiency is reduced after continued usage. Due to the therapeutic effects, high popularity and good nutrient profile of medicinal herbs and plant‐based foods, they have gained remarkable attention recently by both consumers and scientific societies (Rouhi‐Boroujeni et al., 2017; Sedighi et al., 2017). It is proven that curcumin is effective in many diseases, including hypercholesterolemia and hypertension (Shome et al., 2016). Hence, curcumin is pretty propitious as an alternate therapeutic compound for hypertension.

A study was done on high‐fructose‐fed lab rats to check curcumin effects on hepatic steatosis and hyperlipidemia. Results indicated the capability of curcumin in treating obesity, lipid derangements, and high‐fructose‐induced fatty liver over intonation of fat metabolism in the liver, as demonstrated by reduced countenance of transcription factors and lipogenic enzymes. Thus, it was proposed that using curcumin can be useful in the management and deterrence of diet‐induced obesity and related difficulties as an adjuvant (Alsamydai & Jaber, 2018).

Hsu et al. (2017) demonstrate the influence of curcumin on hypertension. According to this study, administration of curcumin in rats with cirrhosis bettered portal hypertension‐related portosystemic collaterals and hemodynamic derangements. It was also reported in the same research study that curcumin reduced splanchnic hyperdynamic flow by reducing mesenteric angiogenesis through vascular endothelial growth factor (VEGF) pathway obstruction and by persuading vasoconstriction via inhibition of endothelial NO (nitric oxide) synthase (eNOS) activation. Likewise, research led by Wang et al. (2017) on lab rats confirmed that curcumin treatment exerted a protecting effect on the retina injury after I/R. Therefore, curcumin can be served as a capable component for hypertensive retinopathy.

Another study was done to check the anti‐hypertension activity of curcumin. In this study, blood lipid profile, glucose tolerance, body composition, metabolic and histopathology characteristics, including left ventricular diastolic stiffness along with histopathology, and systolic blood pressure were all examined. Plasma curcumin concentrations were also measured. These nanoparticles of curcumin displayed parallel improvements in cardiovascular function and exhibited anti‐hypertension potential. Recently, in vivo study was done on rats to discover curcumin protective effect on pulmonary arterial hypertension (PAH). The results showed that curcumin efficiently reduced the right/left ventricle weight ratio and pulmonary artery pressure, as well as narrowing of vessels cavities and alleviating the thickening of vessel walls of pulmonary arteries in lab rats. Thus, the propagation of cells was significantly repressed . Hence, it is proved that curcumin can constrain the propagation of pulmonary artery smooth muscle and protect the pulmonary arteries of MCT‐induced PHA in rats (Guo et al., 2020).

To assess the measurable outcome of supplementation of turmeric/curcumin on blood pressure (BP), Hadi et al. (2019) conducted a meta‐analysis and systematic review. The model used to examine the influence of combined trials was random‐effects model. Improvement in systolic blood pressure (SBP) was observed when turmeric was administered for long time. Kruengtip et al. (2013) conducted a study on usages of curcumin and its derivatives in the illness of pulmonic hypertension. The findings of this study reinforced that curcumin downregulates hypertension by persuaded reduction of endothelium tissue of lab rat pulmonary artery.

Research of Kukongviriyapan et al. (2014) discovered that curcumin has the prospective to be used as treatment agent in oxidative stress and hypertension, which were produced by cadmium (Cd) in laboratory rats. Chelating and anti‐oxidant properties of curcumin are the cause of such effects. This research supported that curcumin has the capability to decrease the systolic blood pressure levels, but it reduces diastolic blood pressure more effectively because of their vasodilatory effect on the arterioles and arteries.

### 4.11 Reproductive role

Sterility or infertility is observed as a communal problem among all societies and cultures. According to an estimate by Greil et al. (2010), it is affecting around 10%–15% couples in reproductive age. Among these infertile couples, about 60% unveil male infertility factors. The directive of spermatogenesis comprises paracrine and endocrine mode of action (Huleihel & Lunenfeld, 2004). Luteinizing hormone (LH) and follicle‐stimulating hormone (FSH) are both the endocrine stimulus of normal spermatogenesis. Previously, it was stated that a lower or higher serum level of both FSH and LH are present in males having azoospermia and infertility (Meeker et al., 2007). Sperm morphology is another critical factor in the male fertility. Khelifa et al. (2014) reported that male reproductive potential is also reduced by anomalies in sperm morphology. It was also revealed in a current report that sperm quality is also affected by lifestyle practices as well as environmental factors (Durairajanayagam, 2018).

An in vivo research was completed by Khalaji et al. (2018) to discover new features of rat's testicular tissue damage induced by compact florescent lamps (CFLs) and to evaluate the influence of curcumin on rat's testis after contact with CFLs. Results of this study exposed that compact florescent lamps meaningfully reduced FSH, testicular weight, and sperm motility levels in serum. The results declared curcumin as an anti‐oxidant substance which has protecting properties on undesirable effects in testicles encouraged by compact florescent lamps.

Azza et al. (2011) observed the influence of curcumin and *Curcuma longa* on hostile reproductive properties encouraged by water nitrate effluence in male mice. At the conclusion of this research, it was found that the lipid profile, comprising triglycerides, total lipid, phospholipids, and total cholesterol, was increased in testicles and serum of lab rats, while dehydroepiandrosterone (DHEA), serum levels of testosterone, epididymal sperm number, DNA, RNA, and total protein contents in testis and serum were decreased in nitrate‐exposed rats. Oguzturk et al. (2012) explored the characteristics of the curcumin in contradiction of acute toxicity induced by cadmium chloride (CdCl_2_) on the reproductive system of male mice. It was observed that cadmium chloride increased oxidative stress significantly. This study discovered that curcumin (1 mg/kg) was helpful in cadmium chloride‐caused oxidative damage and induced anti‐oxidant properties too. A substantial reduction in the motility of sperm and its concentration were observed in the group exposed to cadmium chloride. Nonetheless, after treatment of curcumin, these defects of sperms were improved. It was also observed that administration of cadmium chloride caused edema, vacuities, and severe inflammatory cell penetration in the space between intestine and hemorrhage. Using curcumin as a treating agent reduced the harshness of histopathological alterations.

It was informed by Sak et al. (2013) that curcumin decreases the oxidative stress markers, which can lessen the ovarian reperfusion/ischemia damage. Biochemical and histopathological aspects have shown better and extra satisfactory consequences of curcumin. Research done by Mohebbati et al. (2017) showed that extracts of *Curcuma longa* increased the progesterone level and inhibited the reduction of estrogen level, which strengthens the view that curcumin has anti‐infertility effect. Additional study led by Akomolafe and Aluko (2020) exposed that curcumin assisted in reducing both the activities of caspase‐3 and inflammatory catalogues as well as increased the quantity and quality of the sperm. Therefore, this may signify a potential adjunct against cyclophosphamide (CPA)‐induced spermatogenic human deficits.

Finally, an in vivo research study of Alizadeh et al. (2018) stated that curcumin can increase the sperm concentration, sperm motility, and total count. Another recent investigation displayed beneficial effects of curcumin on fertility rate and semen quality indices by increasing the levels of curcumin dietary supplementation (Kazemizadeh et al., 2019).

### 4.12 Skin curative

The largest organ of our body is the skin, which receives sensory stimuli, maintains the body temperature as well as covers, and separates and protect the body from the exterior environment. Some extrinsic factors and individual's lifestyle choice like solar radiation exposure, smoking, low air moisture, high alcohol intake, and poor diet as well as some diseases like diabetes mellitus are linked with the premature aging of the skin (Panahi et al., 2019). According to many researches, curcumin holds noteworthy beneficial influences for numerous skin conditions, including ultraviolet (UV) protection, anti‐inflammatory attributes, anti‐oxidant effects, chemotherapeutic and chemo‐preventive activity, anti‐microbial effects, and wound‐healing assistances (Jiang et al., 2015; Krausz et al., 2015; Lelli et al., 2017; Li, Gao, et al., 2016; Li, Larregieu, et al., 2016; Toden et al., 2015; Xie et al., 2015). In Table 3, some evidences of curcumins as skin curative are also given.

**TABLE 3:** Curcumin's effects on skin diseases.

Applying estate	Assessment model	Formulation	Interval of contact	Experimental design	References
Skin cancer treatment	Mice	0.021% weight/weight (oral)	1–14 weeks	In the living body	Kim et al. (2014)
	SKH‐1 hairless mouse	15.1 mg/100 μL (topical and oral)	–	In the living body	Phillips et al. (2013)
	Swiss albino mice	3.151 ± 0.0861 drug loading; topical	7 weeks	In the living body	Agrawal et al. (2015)
Wound healing	Breastfeeding women with lactational mastitis	cream made with turmeric (200.1 mg per pump; topical)	72 h	Clinical	Afshariani et al. (2014)
	Mice	Quercetin and curcumin‐loaded phospholipid liposome nanovesicles at 10.11 mg/mL	1 day, 4 days	In vitro and in vitro diagnostics	Castangia et al. (2014)
	Rat	2 .1% concentration curcumin ointment (topical)	21 days	In the living body	Mehrabani et al. (2015)
Psoriasis treatment	Mouse	10.1 μM of curcumin (oral)	20 days	In the living body	Kang et al. (2016)
	Mice	Curcumin nanoparticles enclosed in poly (lactic acid/glycolic acid) (topical)	7 days	In the living body	Sun et al. (2017)
Acne treatment	Mouse	100.1 mg/kg curcumin (oral)	20 days	In the living body	Jagetia and Rajanikant (2015)

It was found by some researchers that curcumin has strong anti‐infectious, anti‐oxidant, and anti‐inflammatory activities that can pose a therapeutic part in the progression of wound healing (Akbik et al., 2014; Mohanty & Sahoo, 2017). Additionally, experimental studies have demonstrated that the curcumin‐treated group significantly outperformed the untreated group in terms of wound healing, epidermal growth rate, and cuticular layer thickness (Kulac et al., 2013; Wen et al., 2012). Nanoparticles of curcumin for healing wounds in persistent cutaneous diseases have been studied in both in vivo and in vitro research and were used by Castangia et al. (2014). The results exhibited that nano‐entrapped curcumin can inhibit the biochemical processes and avert the formation of skin lesions, which usually lead to epithelial injury.

Psoriasis is known to be an auto‐immune dermal and epidermal hyper‐proliferative chronic inflammatory disease, which is instigated by immunologic and genetic aspects and generally has impact in the joints and skin (Lowes et al., 2014). Research by Sun et al. (2017) has suggested that the anti‐inflammatory potential of curcumin could lead it to become an anti‐psoriasis agent. Several reports on the curcumin's inhibitory activities have suggested that its action on the potassium channel (Kv1.3) in T cells plays a vital role in psoriasis. A study accompanied by Kang et al. (2016) on rats suffering from psoriasis‐like diseases exhibited the formation of T‐cell inflammatory factors, like IFN‐γ, IL‐17, TNF‐α, IL‐22, IL‐2, and IL‐8, and which were reduced by 30%–60% after 3 weeks of oral curcumin administration. Likewise, curcumin's anti‐infective effects on the skin have also been described by numerous investigators (Afshariani et al., 2014; Izui et al., 2016; Krausz et al., 2015; Tortik et al., 2016).

## 5 CONCLUSIONS AND FUTURE PERSPECTIVE

Curcumin is an extremely pleiotropic agent, which has been employed for its advantageous influence as customary medication in most nations for centuries. The curcumin pharmacological applications are rapidly rising, developing, and growing creativity, as proved by the researches mentioned in this review article and various further being testified each day. Curcumin is naturally enhanced with a number of useful phytoconstituents, the value of which has been demonstrated in both clinical and experimental studies. Curcumin is a very promising prospective beneficial agent with anti‐cancer, anti‐diabetic, anti‐hypertensive, anti‐microbial, and anti‐inflammatory possessions. It gives reasonable therapeutic retort against various human chronic ailments like cancer, hyperglycemia, high blood pressure, scleroderma, and psoriasis. It has also been proven beneficial in contradiction of diabetes‐induced complications, that is, diabetic nephropathy, diabetic neuropathy, vascular diseases, and diabetes‐linked foot ulcer complications. These useful effects are finest attained when curcumin is joined with some other agents like piperine and carbohydrates, which significantly raise its bioavailability. Overall, it can also be concluded from the above‐defined researches that curcumin is an extremely unrestrained agent and can be utilized as a prime component to plan and produce novel compounds which can assist well in therapeutics prospectively. Additionally, it can also be stated that a comparatively adequate amount can deliver health assistances for people who have not been diagnosed with health conditions.

Finally, it can be demonstrated that adopting nanotechnology, such as nanogels, phospholipid‐containing liposomes, nanostructured lipid carriers, polymeric micelles, nano‐emulsions, and other polymeric nano‐particulates approaches, can overcome the constraints of low stability and deprived biological availability of curcumin. However, supplementary studies are obligatory to effusively explain curcumin's benefits.

## AUTHOR CONTRIBUTIONS

**Hudda Ayub:** Writing – original draft (equal). **Mahad Islam:** Writing – original draft (equal). **Munnaza Saeed:** Resources (equal); writing – original draft (equal). **Husnat Ahmad:** Software (equal); validation (equal); visualization (equal). **Fahad Al‐Asmari:** Conceptualization (equal); funding acquisition (equal). **Mohamed Fawzy Ramadan:** Conceptualization (equal); funding acquisition (equal); writing – original draft (equal); writing – review and editing (equal). **Mohammed Alissa:** Conceptualization (equal); funding acquisition (equal). **Muhammad Adnan Arif:** Software (equal); validation (equal); visualization (equal). **Muhammad Umair Jamil Rana:** Software (equal). **Muhammad Subtain:** Software (equal); validation (equal); visualization (equal). **Muhammad Abdul Rahim:** Writing – review and editing (equal). **Eliasse Zongo:** Conceptualization (equal); funding acquisition (equal). **Nazir Ahmad:** Software (equal); validation (equal); visualization (equal).

## FUNDING INFORMATION

This research was funded by the Deanship of Scientific Research (DSR) at King Faisal University under project no. [KFU241743].

## CONFLICT OF INTEREST STATEMENT

The authors declare no conflict of interest.

## Data Availability

Data are contained within the article.
